# Description and phylogeny of a new species of *Liolaemus* (Iguania: Liolaemidae) endemic to the south of the Plurinational State of Bolivia

**DOI:** 10.1371/journal.pone.0225815

**Published:** 2019-12-02

**Authors:** Cristian Simón Abdala, Alvaro J. Aguilar-Kirigin, Romina Valeria Semhan, Ana Lucia Bulacios Arroyo, Julián Valdes, Marcos Maximiliano Paz, Roberto Gutiérrez Poblete, Pablo Valladares Faundez, Robert Langstroth, James Aparicio

**Affiliations:** 1 Consejo Nacional de Investigación Científicas y Técnicas (CONICET)—Unidad Ejecutora Lillo (UEL), San Miguel de Tucumán, Argentina; 2 Facultad de Ciencias Naturales e Instituto Miguel Lillo (IML), Universidad Nacional de Tucumán, San Miguel de Tucumán, Argentina; 3 Área de Herpetología, Colección Boliviana de Fauna, Campus Universitario de Cota Cota, Facultad de Ciencias Puras y Naturales, Universidad Mayor de San Andrés, La Paz, Bolivia; 4 Cátedra Genética Evolutiva, Facultad de Ciencias Exactas y Naturales y Agrimensura, Universidad Nacional del Nordeste, Corrientes, Argentina; 5 Museo de Historia Natural, Universidad Nacional de San Agustín, Arequipa, Perú; 6 Laboratorio de Zoología Integrativa, Departamento de Biología, Facultad de Ciencias, Universidad de Tarapacá, Arica, Chile; 7 Museo Nacional de Historia Natural (MNHN), Cota Cota (Ovidio Suárez), La Paz, Bolivia; Nanjing Agricultural University, CHINA

## Abstract

The *Liolaemus montanus* group is a diverse group of lizards that ranges from central Peru to southwestern Mendoza, Argentina, including much of the Plurinational State of Bolivia (“Bolivia”) and Chile. The species of this group mainly inhabit high elevation areas with cold temperatures. In the last years, several species of this group have been described, mostly in Argentina and Chile. In Bolivia, there are at least thirteen valid species belonging to the *L*. *montanus* group. In this study, we describe a new species of the *L*. *montanus* group with a marked endemism in the Cordillera de Sama of the Tarija Department, Bolivia, and a combination of unique character states that allows its formal description as a new species. The phylogenetic relationships based on analysis of 159 morphological characters suggest that it belongs to the *L*. *montanus* group, and that it is closest to *Liolaemus pulcherrimus*, which is found allopatrically in a small area of the Jujuy Province, Argentina. The multivariate analyses of 66 morphological characters support the phylogenetic relationships. Statistical analyses of inter-species comparisons of morphological characters are not considered the only methods due to the non-independence of some characters states among species; thus, a phylogenetic analysis is recommended. The detailed revision of specimens of the *L*. *montanus* group held in the collections of Bolivia is filling major geographic gaps and improving our understanding of the phylogenetic and biogeographic relationships of this widely distributed group of South American lizards.

## Introduction

The genus *Liolaemus* includes more than 260 valid species [1, 2, 3, 4, 5, 6], of small to medium-sized lizards distributed from Tierra del Fuego to the Ancash Region of Peru and is the second most speciose amniote clade at the global level [3, 7], surpassed only by *Anolis*. In the last 15 years, its known species richness has increased considerably [3], with ongoing systematic revisions that have resolved many taxonomic conflicts, in certain cases after more than a century [8, 9, 10, 11, 12, 13] and have allowed the description of new species within the genus [5, 13, 14, 15, 16, 17, 18]. The subgenus *Eulaemus* is divided into three large monophyletic groups: the clade comprising the *Liolaemus archeforus*-*kingii* and *Liolaemus lineomaculatus* groups [19]; the *Liolaemus boulengeri* group (characterized by the presence of a patch of enlarged scales in the posterior mid region of the thigh) [9, 20]; and the *L*. *montanus* group, characterized by equal sized scales in the mid posterior region of the thigh [21, 22]. The latter group is composed of more than 60 species [3], found mainly at high altitudes in the Andes of Argentina, Bolivia, Chile and Peru. The *L*. *montanus* group was proposed by Etheridge [21] and is characterized by the presence of a blade-like process of the tibia, associated with hypertrophy of the *tibialis anterior* muscle. In an informal phylogenetic proposal Lobo et al. [1], identified two groups within the *L*. *montanus* group: the *Liolaemus andinus* and *Liolaemus dorbignyi* groups. For many years and by many authors, the identity, distribution and diagnosis of several of these species were mistaken [23, 24, 25, 26, 27, 28].

Recent field studies carried out in the highlands of Argentina, Bolivia, Chile and Peru, as well as revisions of type material and additional specimens from the type localities, have helped clarify the taxonomy of the species related to the *L*. *montanus* group [10, 12, 26, 27, 28, 29]. Despite the wide distribution of *Liolaemus* in Bolivia, their taxonomy and phylogenetic relationships have remained virtually unknown. This lack of information is likely reflected in the currently known species richness in Bolivia, with only 21 valid species to date, of which 14 belong *L*. *montanus* group Table 1: *Liolaemus chlorostictus*, *Liolaemus erguetae*, *Liolaemus fittkaui*, *Liolaemus forsteri*, *Liolaemus islugensis*, *Liolaemus jamesi*, *Liolaemus orientalis*, *Liolaemus pachecoi*, *Liolaemus pantherinus*, *Liolaemus pleopholis*, *Liolaemus puritamensis*, *Liolaemus schmidti*, *Liolaemus signifer*, and, the new species described in this study. Nine *Liolaemus* species have been cited from the Department of Tarija: *Liolaemus chaltin*, *Liolaemus puna* and *Liolaemus variegatus* (subgenus *Liolaemus sensu stricto*); *Liolaemus chacoensis*, *Liolaemus ornatus*, and *Liolaemus simonsii* (*L*. *boulengeri* group); and *L*. *islugensis*, *L*. *orientalis* and *L*. *pantherinus* (*L*. *montanus* group) [30, 31, 32, 33, 34, 35]. However, the number of species has varied; for example, *L*. *simonsii* is considered to be a junior synonym of *L*. *ornatus* [36]. Ongoing revisions of Bolivian material since 2011 by the present authors led to the finding that the lizards from the Tarija Department identified as *L*. *islugensis* by Tarifa et al. [32] and Quinteros and Abdala [34] are in fact quite distinct from that species and that a species new to science might be at hand.

**Table 1:** Species of the *L*. *montanus* group described or cited from Bolivia.

SPECIES	AUTHOR	TAXONOMIC STATUS	DISTRIBUTION
*Liolaemus bolivianus*	Pellegrin, 1909	Junior synonym of *Liolaemus multiformis* (Hellmich, 1962) and by extension *L*. *signifer* (Laurent, 1992)	Bolivia: La Paz: Chililaya (Puerto Pérez)
*L*. *chlorostictus*	Laurent, 1991	Valid	Argentina: Jujuy Bolivia: Potosí
*L*. *erguetae*	Laurent, 1995	Valid	Bolivia: Potosí Chile: Second Region
*L*. *fittkaui*	Laurent, 1986	Valid	Bolivia: Cochabamba: Laguna San Isidro, Laguna Robada, Laguna de Sallamani, Cerro Rodeo, Cotani, Quewiñacocha, Pojo
*L*. *forsteri*	Laurent, 1982	Valid	Bolivia: La Paz: Alto Achachicala, Alto de Animas, Chacaltaya, Charquini, Choquekhota, Chuquiaguillo, El Alto, Hampaturi, Huni, Milluni, Pampalarama, Pinaya, Ovejuyo. Lowlands of Wila Khalani, Hurmutani and Chojña Khota lagoons, headwaters of Orko Jahuira river
*L*. *islugensis*	Ortiz and Marquet, 1987	Valid: Junior synonym of *L*. *pantherinus* (Pincheira-Donsoso and Núñez, 2002)	Bolivia: Oruro, Potosí Chile: First and Second Regions
*L*. *jamesi*	Boulenger, 1891	Valid	Bolivia: Oruro Chile: Fifteenth and First Regions
*Liolaemus lenzi*	Boettger, 1891	Junior synonym of *L*. *multiformis* (Burt and Burt, 1931) and by extension *L*. *signifer* (Laurent, 1992)	Bolivia: La Paz: Lake Titicaca
*L*. *multiformis*	(Cope, 1875)	Junior synonym of *L*. *signifer* (Halloy and Laurent, 1988)	Bolivia: La Paz: Lake Titicaca Peru: Puno
*L*. *orientalis*	Müller, 1924	Valid	Argentina: Jujuy Bolivia: Chuquisaca, Potosí, Tarija
*L*. *pachecoi*	Laurent, 1995	Valid	Bolivia: Potosí Chile: First Region: Quebrada del Inca and Puquios
*L*. *pantherinus*	Pellegrin, 1909	Junior synonym of *L*. *signifer* (Laurent, 1992); valid senior synonym of *L*. *islugensis* (Pincheira-Donoso and Núñez, 2002)	Bolivia: Presumed from vicinity of Lake Titicaca. Cochabamba, Oruro, Tarija (its distribution requires confirmation) Chile: First and Second Regions Peru: Presumed from vicinity of Lake Titicaca
*L*. *pleopholis*	Laurent, 1998	Valid	Bolivia: Oruro: Localities: Sajama, Junthuma, Quilhuiri, Cosapa Chile: Fifteenth Region: Pampa Chucuyo
*L*. *puritamensis*	Núñez and Fox, 1989	Valid	Argentina: Jujuy: Rinconada Bolivia: Potosí: Khastor, Río Blanco, Quetena Chico Chile: Second Region
*L*. *schmidti*	Marx, 1960	Valid	Bolivia: Potosí Chile: First and Second Region
*L*. *signifer*	Duméril and Bibron, 1837	Valid	Bolivia: La Paz, Oruro Peru: Puno
*Liolaemus variabilis* var. *crequii* var. *neveui* var. *courtyi*	Pellegrin, 1909	Junior synonym of *L*. *multiformis* (Hellmich, 1962) and by extension *L*. *signifer* (Laurent, 1992)	Bolivia: La Paz: Tiahuanaco

Table 1. Species of the *L*. *montanus* group described or cited from Bolivia; current taxonomic status and distribution by country and first-order administrative divisions, including the known distribution of the species in neighboring countries. The specimens examined are listed in the Appendix with their respective acronyms.

To validate the species described in this study, we used the general or unified definition of species by de Queiroz [37, 38], who defines a species as an entity that represents independent historic linages, or divergent linages of meta-populations. The use of an a priori diagnostic criterion to test the boundaries among species as a hypothesis that could be empirically accepted or rejected is recommended by some authors [39, 40, 41, 42, 43]; however, with the exception of Aguilar-Kirigin [33], this approach has not been previously applied in taxonomic studies of the *L*. *montanus* group in Bolivia. Our operational criteria for inferring species limits are based on phylogenetic trees, morphological characters, and consideration of the geographic isolation of the new species in the Tajzara Basin of Tarija. We analyzed morphological characters traditionally used in taxonomic studies of *Liolaemus* and performed morphological phylogenetic analyses of 159 characters with 31 terminals to determine the phylogenetic relationships of the new species. We also performed multivariate analyses of 66 characters to evaluate morphological differences among phylogenetically-close species. The phylogenetic analyses indicate that the new species belongs to the *L*. *montanus* group and, within this, that it belongs to a sub-clade including *L*. *fittkaui* from central Bolivia and *Liolaemus griseus*, *L*. *huacahuasicus*, *L*. *montanus*, *Liolaemus orko*, and *L*. *pulcherrimus* from northern Argentina. This new species can be differentiated from the rest of the species of the genus by a combination of morphological characters and color patterns. Following several authors [5, 44, 45, 46] and according to the general concept of species, the new population exhibits a sharp endemism and unique morphological traits that allow distinguishing it as a new species of *Liolaemus*. In this study, we present a new species of the *L*. *montanus* group that had originally been catalogued as *L*. *islugensis* in the Colección Boliviana de Fauna from La Paz, Bolivia [32, 34]; and as *L*. *signifer* in the herpetological collection of Fundación Miguel Lillo, Tucumán, Argentina. This new taxon is endemic to the endorheic basin of Tajzara in the Reserva Biológica Cordillera de Sama of the Tarija Department of Bolivia.

## Materials and methods

Examined material is listed in the Appendix. All specimens were collected by hand or noose. All revised specimens are fixed in 10% formalin, and preserved them in 80% ethanol, and deposited them in Colección Boliviana de Fauna (CBF), La Paz, Bolivia, and Fundación Miguel Lillo (FML), San Miguel de Tucumán, Argentina. This study was carried out in strict accordance with the recommendations in the guide for care and use of animals of both institutions and all efforts were made to minimize suffering. This study was approved by Ministerio de Medio Ambiente, Viceministerio de Medio Ambiente, Biodiversidad y Cambios Climáticos as well as Servicio Nacional de Áreas Protegidas (SERNAP), La Paz, Plurinational State of Bolivia.

Characters commonly used in *Liolaemus*, described and cited mainly by Laurent [47], Etheridge [21, 48], Abdala [9], Abdala and Juárez [13], and Gutiérrez et al. [6] were studied. Color in life was described based on field observations and photographs of captured specimens. Squamation was examined under a binocular microscope, and body measurements were taken with a ± 0.01 mm precision caliper. Neck fold terminology follows Abdala [9], whereas body color pattern terminology follows Lobo and Espinoza [49], Abdala [9] and, Gutiérrez et al. [6].

Each morphometric variable was measured three times on the same individual, and the mean value for each species was used in the subsequent analyses. Only adult males were used in the multivariate analysis to avoid confounding effects of intraspecific allometric variation [50] and to elude confusions in the multivariate analyses due to possible sexual dimorphism. All bilateral characters were measured on the right side. The measured morphometric traits were: snout-vent length (SVL); minimum distance between the nasal scales (DN); snout width at the edge of the canthal scale (AH); distance from the nose to the back edge of the canthal scale (NC); distance between the posterior edge of the superciliary series (EO); length of the interparietal (LEI); length of the parietal (PA); mental scale width (AM); length of the mental scale (LM); distance from nostril to mouth (NB); rostral height (HR); length of the subocular scale (ES); auditory meatus height (hTy); auditory meatus width (aTy); length of the preocular scale (LPO); preocular width (LPOT); length of the fourth supralabial scale (LCSP); length of the fourth lorilabial scale (LCLB); length between orbits (DEO); length of the first finger of the forelimb, without claw (1D); length of the claw of the fourth finger of the forelimb (G4D); length of the fifth finger of the forelimb without claw (5D); humerus width (AHU); distance from the insertion of the forelimb in the body toward the elbow (LEA1); thigh width (AMU); length of the first toe of the hind limb without claw (1P); length of the claw of the fourth toe of the hind limb (4U); length of the five dorsal scales in a row in the middle of the body (ED); cloacal opening width, measured distance between the corners of the cloaca (PP); body width (AL); width of the base of the tail (WTB); upper width of the pygal area (ASPI); length of the pygal area (LPI).

The following meristic characters were counted: number of scales around the interparietal scale (A11); number of supralabials on the right side (A12); number of supralabials on the left side (A15); number of infralabials on the right side (A13); number of infralabials on the left side (A19); number of scales around the mental scale (A14); number of scales around the rostral scale (A16); number of lorilabials (A17–1); Hellmich index (A18); subdigital lamellae of the first finger of the forelimb (A20–1); subdigital lamellae of the second finger of the forelimb (A20–2); subdigital lamellae of the third finger of the forelimb (A20–3); subdigital lamellae of the fourth finger of the forelimb (A20–4); subdigital lamellae of the fifth finger of the forelimb (A20–5); subdigital lamellae of the first toe of the hind limb (A21–1); subdigital lamellae of the second toe of the hind limb (A21–2); subdigital lamellae of the third toe of the hind limb (A21–3); subdigital lamellae of the fourth toe of the hind limb (A21–4); subdigital lamellae of the fifth toe of the hind limb (A21–5); number of dorsal scales between the occiput and the level of the anterior edge of the thigh (A22); number of precloacal pores (A26); number of scales between canthal and nasal scales (M2); number of scales around the nasal scale (M3); number of supraocular enlarged scales in the right side (M5); number of supraocular enlarged scales in the left side (M4); number of organs in the postrostral scales. The scale organs are present on head scales of *Liolaemus* species and, appear to be randomly distributed to each individual examined (M13); number of organs in the third lorilabial scale (M14); number of organs in the scale above the row of the lorilabial scales and below the canthal and preocular scales (M15); number of gular scales (M23); number of scales around midbody (M26); number of ventral scales (M32); number of auricular scales, projecting scales on anterior edge of auditory meatus (M34); and number of paravertebral spots in the right side (D6).

### Phylogenetic analyses

Phylogenetic analyses were performed with the morphological matrix of Gutiérrez et al. [6], which includes 159 characters and 31 terminals (*Ctenoblepharys adspersa* and *Phymaturus palluma* as the outgroup and 29 terminals of the *L*. *montanus* group). External morphological data to build the matrix were taken from preserved museum specimens, see supplementary material in http://morphobank.org/permalink/?P3206. In the phylogenetic analysis, the parsimony criterion was used as the optimality criterion, selecting only shorter trees, or those with fewer homoplasies. The software used to search for phylogenetic hypotheses was TNT 1.5 (Tree Analysis Using New Technology, version 1.0) [51]. Discrete characters were classified into binary polymorphic, binary non-polymorphic, multistate polymorphic, and multistate non-polymorphic. The binary and multistate polymorphic characters were treated as given by Wiens [52]. Continuous characters were analyzed using the methodology proposed by Goloboff et al. [53], and these were “standardized” using an associated script (mkstandb.run). Heuristic searches were made to find the most parsimonious trees. Tree bisection and reconnection (TBR) was used for branch permutation. The matrix was analyzed using the “implied weights” method [54]. Twenty-one runs were made with the evidence: matrix one run was made with equally weights (EW) and the other 20 were made with implied weights (IW), with K values ranging from three to 22. One thousand five hundred replicates were run with each K value. Group support was estimated using symmetric resampling, a method that is not distorted by differential costs [51], with 1000 replicates and a 0.33 deletion probability, and the support values go from zero to 100.

### Statistical analysis

Normal distributions of the morphometric data were examined using the Kolmogorov-Smirnov test (P ≤ 0.05), and homoscedasticity was evaluated with Levene’s test. To reduce the effect of non-normal distributions of the morphological data, all continuous variables were log_10_ transformed and meristic variables were square root transformed [55, 56, 57]. All operational taxonomic units were analyzed by two distinct treatments. A principal component analysis (PCA) was employed to analyze the morphological variation and discriminant function analyses (DFA) were used to verify morphological variation between and within each *Liolaemus* species employing a jackknife classification matrix [58, 59, 60, 61]. Four species distributed in Argentina (*L*. *griseus*, *L*. *huacahuasicus*, *L*. *orko*, and *L*. *pulcherrimus*) and the new species from Bolivia, were used as comparative groups for building the PCA and the DFA. *Liolaemus fittkaui* was not included in the multivariate analyses due to the availability of only two specimens in the Fundación Miguel Lillo collections; however, it was included in the phylogenetic analyses.

The PCA analysis was performed to evaluate the distribution of individuals corresponding to the five species (*L*. *griseus*, *L*. *huacahuasicus*, *L*. *orko*, *L*. *pulcherrimus* and, *Liolaemus* sp. nov.) in the multivariate space. The PCA was based on the correlation matrices of the morphological variables to reduce dimensionality of the data [60, 62]. The PCA and DFA were evaluated separately for continuous and meristic characters, following the recommendations of certain authors to not joint both matrices in multivariate analyses, although there is no mathematical consensus on this approach [63]. Once the PCA was performed, and the lineal combinations that explained the highest variation were extracted, whether the new species exhibited similar or different morphological characters was examined by means of a DFA, with defined *Liolaemus* groups based on the results of the PCA. This mathematical model allows assessing whether the groups discriminated by the DFA correspond to those established by the PCA. The DFA produces a linear combination of variables that maximizes the probability of correctly assigning observations to predetermined groups; and simultaneously, new observations can be classified in one of the groups, providing likelihood values of such classification [63, 64]. All statistical analyses were performed using the Statistica software, version 7.0 [65].

### Nomenclatural acts

The electronic edition of this article conforms to the requirements of the amended International Code of Zoological Nomenclature, and hence the new names contained herein are available under that Code from the electronic edition of this article. This published work and the nomenclatural acts it contains have been registered in ZooBank, the online registration system for the ICZN. The ZooBank LSIDs (Life Science Identifiers) can be resolved and the associated information viewed through any standard web browser by appending the LSID to the prefix “http://zoobank.org/”. The LSID for this publication is: urn:lsid:zoobank.org:author:3528E2D9-DB5E-4314-8672-F9751BB52FFC. The electronic edition of this work was published in a journal with an ISSN, and has been archived and is available from the following digital repositories: PubMed Central, LOCKSS.

## Results

### Phylogenetic analysis

**Fig 1:**
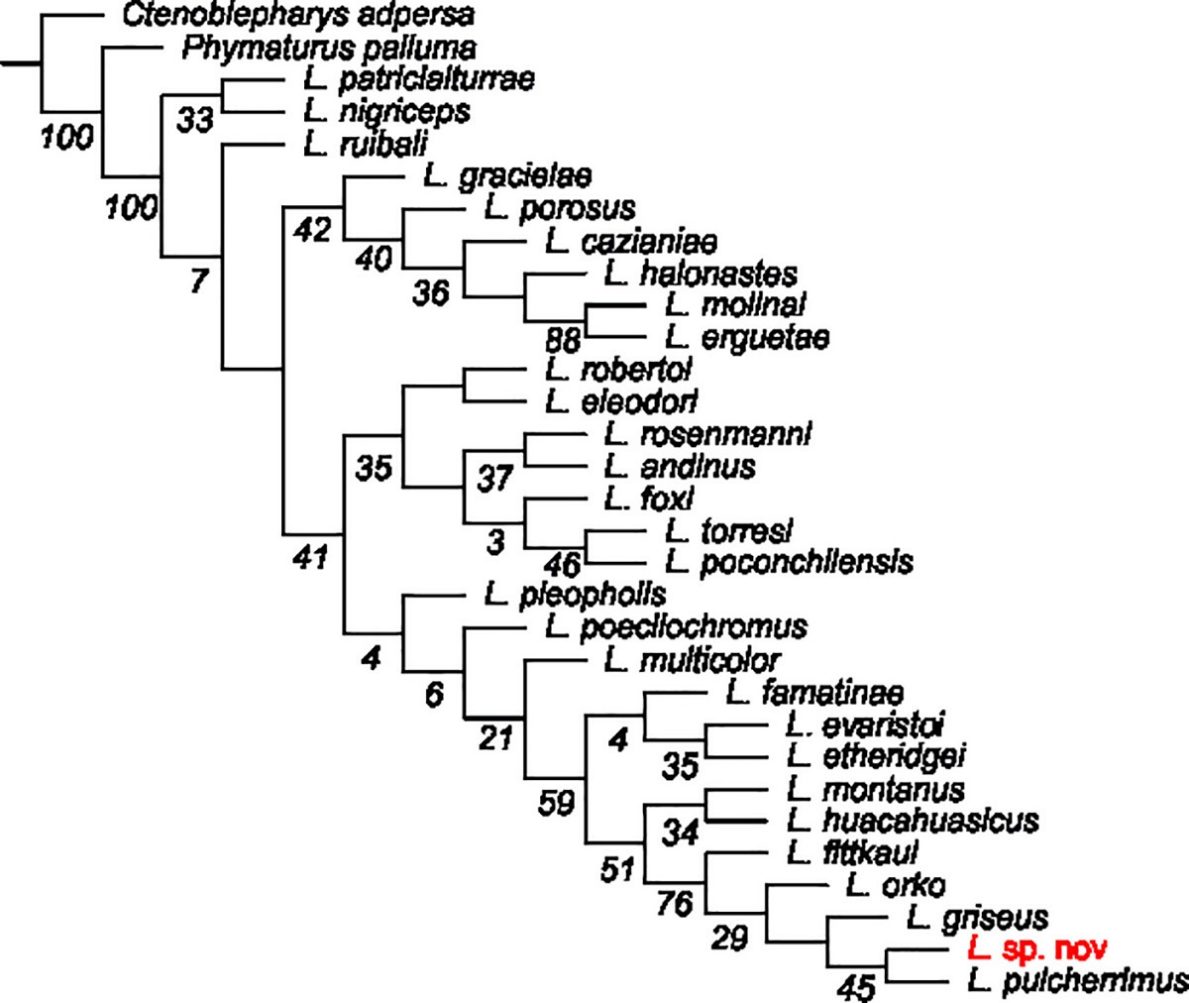
Phylogenetic tree showing the relationships between *Liolaemus* sp. nov., within the *L*. *montanus* group. The values correspond to the support measure, symmetric resampling.

The morphological phylogenetic hypothesis indicates that *Liolaemus* sp. nov., belongs to the *L*. *montanus* group, together with *Liolaemus etheridgei*, *Liolaemus evaristoi*, *Liolaemus famatinae*, *L*. *fittkaui*, *L*. *griseus*, *L*. *huacahuasicus*, *L*. *montanus*, *L*. *orko* and, *L*. *pulcherrimus* (Fig 1). This clade is supported by 10 synapomorphies, of which six are continuous (fewer scales around midbody, dorsals, neck scales, ventrals, pygals, and weaker correlation between tibial length and SVL in respect to other species of *L*. *andinus* group) and four are discrete (scales of the body, dorsum, laminar, triangular, keeled, and imbricate). The clade (*L*. *fittkaui* (*L*. *orko* (*L*. *griseus* (*L*. *pulcherrimus* + *Liolaemus* sp. nov.)))) supported by three characters: greater number of temporal scales and presence of parallel light and dark spots from the eye to neck in the temporal region has good support (Fig 1) and is inferred by the analyses with K = 11 to 22, while the relationship between *L*. *pulcherrimus* as sister species of *Liolaemus* sp. nov., is inferred in all the analyses performed with the implied weights with five synapomorphies: greater number of number of scales separating the fourth chinshields, greater number of gulars, higher ratio of femur length/SVL, suprascapular and gular folds present. In the trees obtained with K = 3 to 10, the (*L*. *pulcherrimus* + *Liolaemus* sp. nov.) clade remains outside the (*L*. *griseus* (*L*. *orko* (*L*. *montanus* + *L*. *huacahuasicus*) + (*L*. *fittkaui* + *L*. *famatinae*))) clade.

*Liolaemus* sp. nov. is supported by 13 autopomorphies in the tree, two continuous: lower ratio of tail length/SVL, higher ratio of femur length/SVL, two discrete scale characters: slight keel in the dorsal scales of the body, precloacal pores absent or few in females, and nine discrete in terms of coloration: head never darker than body color and absence of dorsolateral bands in males, and in both sexes, paravertebral and lateral blotches presenting dark ocelli with light centers; pattern and colors of the hands and feet brindled.

### Statistical analysis

The summary statistics for all the non-transformed continuous and meristic characters taken from five species of *Liolaemus* are shown in Table 2.

**Table 2:** Morphological characteristics used in this study for statistical analysis.

Morphological Character	*L*. *griseus* n = 10	*L*. *huacahuasicus* n = 15	*L*. *orko* n = 10	*L*. *pulcherrimus* n = 12	*Liolaemus* sp. nov. n = 15
SVL	54.39–69.37 62.29 ± 4.93	63.58–76.03 69.59 ± 4.35	54.01–70.68 63.13 ± 5.87	65.94–73.17 68.19 ± 2.24	62.26–71.87 66.96 ± 3.23
DN	2.01–2.69 2.29 ± 0.26	2.21–2.90 2.56 ± 0.19	2.12–2.74 2.43 ± 0.26	2.20–2.51 2.41 ± 0.09	1.39–1.77 1.58 ± 0.13
AH	4.09–4.68 4.42 ± 0.19	4.27–5.19 4.75 ± 0.31	4.65–5.58 5.13 ± 0.42	4.65–5.49 5.09 ± 0.30	4.86–5.97 5.33 ± 0.29
NC	4.09–4.74 4.47 ± 0.27	4.74–5.36 5.04 ± 0.17	3.81–4.98 4.45 ± 0.48	4.70–4.99 4.86 ± 0.09	2.59–3.50 3.01 ± 0.23
EO	6.68–7.19 6.90 ± 0.19	7.22–7.81 7.55 ± 0.18	7.01–7.55 7.31 ± 0.21	7.28–8.21 7.77 ± 0.33	7.51–8.80 8.28 ± 0.33
LEI	1.02–1.46 1.23 ± 0.15	1.03–1.57 1.29 ± 0.18	1.67–2.54 2.12 ± 0.35	1.35–1.94 1.69 ± 0.19	0.92–1.97 1.49 ±0.32
PA	1.46–1.67 1.57 ± 0.07	1.59–1.99 1.79 ± 0.15	1.55–2.24 1.89 ± 0.29	1.14–1.84 1.55 ± 0.17	1.22–2.00 1.65 ± 0.28
AM	1.85–2.58 2.15 ± 0.23	2.42–3.15 2.78 ± 0.25	2.27–2.96 2.58 ± 0.26	2.45–3.05 2.77 ± 0.24	1.51–1.99 1.77 ± 0.16
LM	1.07–1.62 1.33 ± 0.21	1.33–1.74 1.47 ± 0.10	1.26–1.55 1.39 ± 0.10	1.43–1.77 1.61 ± 0.12	2.60–3.36 3.08 ± 0.20
NB	1.23–1.73 1.43 ± 0.16	1.34–1.66 1.51 ± 0.12	1.31–1.69 1.49 ± 0.14	1.51–1.89 1.72 ± 0.12	1.49–1.86 1.69 ± 0.12
HR	0.81–1.09 1.00 ± 0.08	0.92–1.16 1.06 ± 0.08	0.86–1.19 1.02 ± 0.12	0.82–1.33 1.06 ± 0.15	1.05–1.37 1.19 ± 0.08
ES	3.70–3.91 3.83 ± 0.07	3.98–4.31 4.13 ± 0.12	3.40–3.94 3.71 ± 0.20	3.90–4.60 4.20 ± 0.26	3.83–5.72 4.43 ± 0.44
hTy	2.24–3.41 2.81 ± 0.43	2.47–4.11 3.22 ± 0.73	2.43–2.96 1.69 ± 0.23	2.47–3.25 2.81 ± 0.21	2.46–3.00 2.69 ± 0.15
aTy	1.07–1.52 1.32 ± 0.14	1.24–1.78 1.53 ± 0.18	1.10–1.85 1.69 ± 0.23	1.17–1.61 1.42 ± 0.15	1.14–1.76 1.49 ± 0.18
LPO	1.27–1.40 1.33 ± 0.04	1.36–1.74 1.55 ± 0.15	1.18–1.47 1.30 ± 0.10	1.28–1.73 1.52 ± 0.13	1.12–1.85 1.48 ± 0.21
LPOT	0.48–0.85 0.67 ± 0.11	0.55–0.82 0.70 ± 0.09	0.54–0.88 0.74 ± 0.12	0.60–0.98 0.81 ± 0.11	0.73–1.52 1.09 ± 0.23
LCSP	1.19–1.75 1.47 ± 0.22	1.00–1.64 1.32 ± 0.18	0.84–1.73 1.26 ± 0.34	1.21–2.21 1.80 ± 0.34	0.99–2.18 1.44 ± 0.33
LCLB	1.07–1.10 1.08 ± 0.01	0.82–1.62 1.15 ± 0.22	0.69–1.33 1.05 ± 0.26	1.00–1.55 1.26 ± 0.18	0.96–1.68 1.27 ± 0.19
DEO	5.52–7.18 6.53 ± 0.49	6.09–7.65 6.96 ± 0.52	6.14–7.19 6.68 ± 0.40	6.17–7.74 7.03 ± 0.45	1.06–1.54 1.34 ± 0.15
1D	2.90–3.54 3.18 ± 0.22	2.75–3.54 3.10 ± 0.23	3.34–4.18 3.84 ± 0.33	2.98–3.63 3.31 ± 0.22	2.45–3.07 2.68 ± 0.14
G4D	1.45–1.98 1.68 ± 0.18	1.52–2.02 1.83 ± 0.17	1.44–2.29 1.95 ± 0.34	1.39–2.05 1.70 ± 0.24	1.31–1.87 1.60 ± 0.15
5D	3.39–3.96 3.52 ± 0.17	3.58–4.33 4.05 ± 0.26	3.01–4.30 3.72 ± 0.53	3.41–4.79 4.08 ± 0.37	2.98–4.02 3.36 ± 0.26
AHU	3.01–3.77 3.44 ± 0.26	3.49–4.44 4.05 ± 0.32	3.15–4.29 3.76 ± 0.47	3.89–5.35 4.53 ± 0.56	3.33–4.96 4.35 ± 0.52
LEA1	6.94–8.90 8.05 ± 0.67	8.09–9.53 8.75 ± 0.47	8.19–9.19 8.73 ± 0.44	8.40–9.76 9.15 ± 0.37	6.67–8.78 7.61 ± 0.65
AMU	3.85–5.04 4.38 ± 0.39	4.12–5.59 4.93 ± 0.42	4.30–5.23 4.73 ± 0.37	5.20–6.94 6.06 ± 0.54	4.96–7.45 6.14 ± 0.71
1P	3.91–4.21 4.04 ± 0.09	3.43–5.14 4.26 ± 0.61	4.24–5.25 4.71 ± 0.36	3.88–6.07 5.08 ± 0.79	3.05–3.58 3.27 ± 0.16
4U	1.46–2.47 1.86 ± 0.31	1.91–2.44 2.20 ± 0.19	1.87–2.39 2.16 ± 0.20	1.72–2.36 2.09 ± 0.19	1.40–1.94 1.62 ± 0.16
ED	4.00–4.39 4.21 ± 0.15	3.28–4.29 3.81 ± 0.37	2.97–3.91 3.45 ± 0.38	3.08–3.67 3.33 ± 0.18	2.92–4.07 3.54 ± 0.41
PP	5.76–7.96 6.78 ± 0.78	3.94–7.34 5.66 ± 1.03	6.04–7.59 6.80 ± 0.67	7.48–10.16 9.04 ± 0.90	7.97–10.19 8.97 ± 0.61
AL	11.87–21.80 16.53 ± 3.20	11.18–19.64 16.11 ± 2.86	11.69–16.38 14.27 ± 1.80	15.69–20.11 17.68 ± 1.34	14.98–23.44 19.06 ± 2.65
WTB	4.47–5.99 5.37 ± 0.52	6.63–12.47 9.39 ± 2.07	6.23–9.40 8.03 ± 1.12	6.75–8.36 7.49 ± 0.65	8.73–11.44 10.03 ± 0.77
ASPI	4.67–7.34 5.73 ± 0.79	4.57–5.45 5.06 ± 0.27	4.87–5.64 5.28 ± 0.28	6.00–7.31 6.80 ± 0.40	5.22–7.31 6.30 ± 0.75
LPI	5.49–8.20 6.64 ± 1.01	6.54–7.67 7.10 ± 0.33	5.78–7.68 6.78 ± 0.84	7.27–8.93 7.86 ± 0.61	7.69–9.99 8.58 ± 0.56
A11	5–7 6.10 ± 0.74	7–8 7.47 ± 0.52	7–9 8.30 ± 0.67	7–9 7.58 ± 0.79	4–8 6.80 ± 1.15
A12	7–11 8.90 ± 1.37	8–10 9.13 ± 0.92	7–9 8.60 ± 0.70	8–9 8.67 ± 0.49	6–9 7.80 ± 0.77
A15	8–10 9.30 ± 0.67	8–10 9.07 ± 0.70	7–9 8.10 ± 0.74	8–9 8.83 ± 0.39	6–8 7.20 ± 0.77
A13	5–7 5.90 ± 0.74	5–7 5.73 ± 0.70	6–7 6.10 ± 0.32	6–8 7.00 ± 0.85	5–6 5.40 ± 0.51
A19	5–7 5.70 ± 0.67	5–6 5.67 ± 0.49	5–7 5.80 ± 0.63	6–9 7.33 ± 1.07	5–7 5.60 ± 0.63
A14	4 4.00 ± 0.00	4–5 4.07 ± 0.26	4–5 4.10 ± 0.32	4 4.00 ± 0.00	4 4.00 ± 0.00
A16	6 6.00 ± 0.00	6 6.00 ± 0.00	6–7 6.40 ± 0.52	6 6.00 ± 0.00	6–7 6.07 ± 0.26
A17–1	7–9 7.90 ± 0.74	8–12 9.73 ± 1.44	8–10 9.00 ± 0.82	9 9.00 ± 0.00	7–9 8.13 ± 0.74
A18	14–17 15.80 ± 1.03	13–18 15.60 ± 1.24	16–18 16.70 ± 0.67	16–19 17.67 ± 1.07	14–20 17.80 ± 1.70
A20–1	7–9 8.30 ± 0.67	8–10 9.40 ± 0.74	9–11 9.80 ± 0.79	7–9 8.08 ± 0.79	7–10 8.53 ± 0.83
A20–2	11–14 12.40 ± 0.97	11–14 12.40 ± 1.18	13–15 13.60 ± 0.70	12–14 13.42 ± 0.79	9–14 12.33 ± 1.18
A20–3	16–18 16.80 ± 0.79	13–18 15.67 ± 1.40	17–19 18.00 ± 0.82	16–17 16.67 ± 0.49	13–17 15.80 ± 1.21
A20–4	17–19 17.70 ± 0.82	15–19 16.53 ± 1.19	18–20 18.90 ± 0.74	17–18 17.50 ± 0.52	13–19 16.67 ± 1.54
A20–5	10–12 11.00 ± 0.67	8–13 10.40 ± 1.30	10–14 12.50 ± 1.35	9–13 11.00 ± 1.21	8–12 10.60 ± 1.06
A21–1	8–11 9.30 ± 0.95	13–16 14.47 ± 0.74	9–11 10.10 ± 0.74	8–11 10.00 ± 1.04	9–11 9.73 ± 0.80
A21–2	11–15 13.70 ± 1.42	13–16 14.47 ± 0.74	14–16 15.00 ± 0.82	13–16 14.58 ± 0.79	12–15 13.33 ± 0.82
A21–3	14–20 17.50 ± 2.12	16–20 17.53 ± 1.25	19–21 19.80 ± 0.79	17–20 18.58 ± 0.90	15–19 17.60 ± 1.30
A21–4	20–26 22.50 ± 2.07	21–24 22.60 ± 1.12	23–26 24.70 ± 1.16	21–25 23.00 ± 1.35	19–26 22.07 ± 1.87
A21–5	13–16 14.50 ± 0.97	14–17 15.33 ± 0.82	15–16 15.80 ± 0.42	14–18 15.75 ± 1.29	11–16 13.73 ± 1.22
A22	56–75 64.00 ± 6.82	60–69 63.40 ± 2.92	71–87 76.90 ± 4.98	86–93 89.50 ± 2.50	69–85 76.20 ± 4.74
A26	6–7 6.30 ± 0.48	5–7 6.33 ± 0.72	5–8 6.50 ± 0.97	5–8 6.92 ± 1.24	3–9 6.80 ± 1.61
M2	2 2.00 ± 0.00	2 2.00 ± 0.00	1–2 1.80 ± 0.42	1–2 1.83 ± 0.39	1–2 1.73 ± 0.46
M3	6 6.00 ± 0.00	6–7 6.67 ± 0.49	6–7 6.60 ± 0.52	6–8 6.58 ± 0.79	5–8 6.27 ± 1.03
M5	3–6 5.00 ± 1.05	4–6 4.87 ± 0.74	4–6 5.10 ± 0.88	5–6 5.83 ± 0.39	5–8 5.93 ± 0.88
M4	5–6 5.20 ± 0.42	4–6 5.20 ± 0.68	4–5 4.50 ± 0.53	4–7 5.50 ± 1.09	5–7 5.60 ± 0.74
M13	3–4 3.60 ± 0.52	3–5 4.07 ± 0.80	3–5 4.20 ± 0.79	2–5 3.67 ± 0.98	4–12 6.00 ± 2.00
M14	2–9 5.20 ± 2.70	3–8 5.27 ± 1.58	6–8 6.80 ± 0.63	5–10 7.58 ± 1.62	3–11 5.93 ± 2.34
M15	5–11 8.30 ± 2.11	3–5 4.27 ± 0.80	3–5 4.40 ± 0.84	3–8 5.83 ± 1.90	3–11 6.27 ± 2.63
M23	30–36 32.90 ± 2.08	29–34 31.73 ± 1.79	31–37 33.40 ± 2.01	31–36 33.92 ± 1.73	23–28 25.80 ± 1.78
M26	59–62 60.50 ± 0.85	56–65 61.07 ± 2.96	60–70 65.70 ± 3.53	70–76 72.50 ± 2.07	66–79 71.13 ± 4.24
M32	59–78 68.60 ± 6.74	68–80 73.07 ± 4.06	71–82 77.50 ± 3.57	71–78 74.42 ± 2.07	73–90 78.60 ± 4.19
M34	1–2 1.70 ± 0.48	2–3 2.47 ± 0.52	2–3 2.50 ± 0.53	1–4 2.25 ± 0.97	2–5 3.27 ± 0.88
D6	9–12 10.10 ± 1.20	8–11 9.27 ± 0.80	10–12 11.20 ± 0.79	10–16 12.92 ± 2.15	7–11 8.80 ± 1.08

Table 2. Morphological characteristics of five species of *Liolaemus* studied in this work. Range in the first line; mean ± standard deviation (mm) for quantitative characters in the second line.

The homogeneity of variance was not supported for either continuous or meristic characters by the Levene’s test in some groups. Therefore, the results of the principal components analyses should be preferred for deriving linear combinations of the variables that summarize the variation in the data set. The results of the PCA for continuous and meristic characters are presented separately in Tables 3 and 4.

**Table 3:** Principal component (PC) axes loadings of continuous characters.

Males	PC1	PC2	PC3	PC4	PC5
Percentage variation accounted for	33.55	20.38	8.53	7.59	4.63
Eigenvalue	11.07	6.72	2.81	2.50	1.53
Snout-vent length	–0.32	0.76	–0.21	–0.36	–0.10
Minimum distance between the nasal scales	0.79	0.53	–0.02	–0.13	0.02
Snout width at the edge of the flake canthal	–0.62	0.50	–0.07	0.40	0.04
Distance from the nose to the back edge of the flake canthal	0.78	0.50	0.08	–0.26	0.07
Distance between the posterior edge of the series superciliary	–0.83	0.27	–0.13	0.03	0.15
Length of the interparietal	0.02	0.47	0.19	0.77	–0.09
Length of the parietal	0.18	0.40	–0.47	0.40	–0.31
Mental flake width	0.59	0.69	–0.03	–0.09	0.25
Length of the mental scale	–0.96	–0.10	–0.10	0.08	–0.04
Distance from nostril to the mouth	–0.57	0.53	0.08	0.14	0.10
Rostral height	–0.64	0.30	–0.26	0.07	–0.22
Length of the subocular scale	–0.67	0.28	–0.04	–0.30	–0.03
Ear height	0.10	0.43	–0.57	–0.35	0.13
Ear width	0.05	0.33	–0.45	0.46	–0.10
Length of the preocular scales	–0.26	0.43	–0.12	–0.32	0.17
Preocular width	–0.81	0.17	0.01	0.13	–0.19
Length of the fourth supralabial flake	–0.19	0.47	0.62	–0.15	–0.27
Length of the fourth lorilabial flake	–0.41	0.34	0.25	–0.14	–0.22
Length between orbits	0.89	0.34	0.13	–0.15	0.12
Length of the first finger of the forelimb, without the claw	0.67	0.35	0.28	0.40	–0.15
Length of the claw of the fourth finger of the forelimb	0.33	0.53	–0.18	0.26	–0.37
Length of the fifth finger of the forelimb; without the claw	0.35	0.70	–0.08	–0.08	0.17
Humerus width	–0.54	0.62	0.11	–0.15	0.15
Distance from the insertion of the forelimb in the body toward the elbow	0.43	0.69	0.22	–0.02	0.11
Thigh width	–0.76	0.36	0.22	–0.03	0.26
Length of the first finger of the hind limb, without the claw	0.57	0.51	0.49	0.16	0.08
Length of the claw of the fourth finger of the hind limb	0.56	0.68	–0.13	0.04	–0.07
Length of five dorsal scales	0.19	0.08	–0.03	–0.43	–0.78
Cloacal opening width	–0.65	0.05	0.59	0.25	0.05
Body width	–0.60	0.27	0.07	–0.40	–0.22
Width of the base of the tail	–0.54	0.35	–0.55	0.16	0.26
Upper width of the pygal area	–0.58	0.26	0.51	–0.05	0.03
Length of the pygal area	–0.79	0.40	0.01	–0.06	–0.05

**Table 4:** Principal component (PC) axes loadings of meristic characters.

Males	PC1	PC2	PC3	PC4	PC5
Percentage variation accounted for	19.75	12.63	9.56	7.19	5.62
Eigenvalue	6.52	4.17	3.15	2.37	1.86
Number of scales around the interparietal scale	0.51	0.02	–0.21	0.36	0.27
Supralabials number on the right side	0.35	–0.43	0.19	0.25	–0.35
Supralabials number on the left side	0.39	–0.56	0.46	0.11	–0.11
Infralabials number on the right side	0.47	0.15	0.60	0.19	–0.08
Infralabials number on the left side	0.32	0.25	0.63	0.23	–0.04
Number of scales around mental scale	0.08	–0.13	–0.13	0.23	–0.39
Number of scales around the rostral scale	0.20	0.07	–0.34	–0.27	0.08
Number of lorilabials	0.33	–0.17	–0.06	0.68	–0.18
Hellmich index	–0.02	0.69	0.11	0.02	–0.01
Subdigital lamellae of the first finger of the forelimb	0.39	–0.29	–0.66	0.05	–0.11
Subdigital lamellae of the second finger of the forelimb	0.73	0.29	–0.03	–0.31	0.01
Subdigital lamellae of the third finger of the forelimb	0.65	0.13	0.01	–0.54	0.05
Subdigital lamellae of the fourth finger of the forelimb	0.67	0.17	0.00	–0.47	0.05
Subdigital lamellae of the fifth finger of the forelimb	0.64	0.15	–0.13	–0.18	0.11
Subdigital lamellae of the first toe of the hind limb	0.32	–0.02	–0.32	0.31	0.30
Subdigital lamellae of the second toe of the hind limb	0.67	–0.21	–0.20	0.22	–0.01
Subdigital lamellae of the third toe of the hind limb	0.71	0.27	–0.22	–0.22	–0.04
Subdigital lamellae of the fourth toe of the hind limb	0.73	0.07	–0.34	–0.18	–0.11
Subdigital lamellae of the fifth toe of the hind limb	0.72	–0.13	–0.01	0.21	–0.15
Number of dorsal scales between the occiput and the level of the anterior edge of the thigh	0.40	0.77	0.21	0.14	–0.06
Precloacal number of pores	0.05	0.18	0.03	0.17	–0.44
Number of scales between canthal and nasal	0.11	–0.26	0.04	0.22	0.74
Number of scales around the nasal scale	0.22	–0.04	–0.10	0.34	0.53
Supraoculars number enlarged scale in the right side	–0.04	0.52	0.12	–0.01	–0.01
Supraoculars number enlarged scale in the left side	–0.29	0.19	0.23	0.10	–0.11
Number of scales between canthal and nasal scales	–0.45	0.42	–0.28	0.21	0.16
Number of organs in the third lorilabial scale	0.08	0.32	0.27	0.21	0.40
Number of organs above the row of lorilabials scales and below the canthal and preocular scales	–0.40	0.13	0.48	–0.31	0.13
Gular number of scales	0.57	–0.36	0.45	–0.01	0.10
Number of scales around the middle body	0.05	0.86	0.11	0.25	–0.07
Number of ventral scales	0.15	0.57	–0.41	0.31	–0.16
Number of auricular scales	–0.28	0.41	–0.39	0.14	–0.03
Number of paravertebral spots in the right side	0.57	0.14	0.37	0.03	0.11

Table 3. Principal component (PC) axes loadings of continuous characters for *L*. *griseus* (n = 10), *L*. *huacahuasicus* (n = 15), *L*. *orko* (n = 10), *L*. *pulcherrimus* (n = 12), and *Liolaemus* sp. nov. (n = 15). Eigenvectors, eigenvalues, and percentage of variance explained for the first two principal components from transformed data in the five putative species of *Liolaemus*.

The first five components of continuous characters explained 74.68% of the variation, and a screen plot test of the PCs indicated that only the two first components contained nontrivial information. The first axis represents morphological variation, loading for most variables negatively and accounting for 33.55% of the variation, with strong loadings for length of the mental scale, length between orbits, distance between the posterior edges of the superciliary series, preocular width, minimum distance between the nasal scales, length of the pygal area, distance from the nose to the back edge of the scale canthal, and thigh width. The second axis represents body size, and accounts for most of the remaining variation, with strong loadings for snout-vent length, and length of the fifth finger of the forelimb without claw. The next axes account for the remaining variation, with important loads for length of the five dorsal scales in a row in the middle of the body, interparietal length, and length of the fourth supralabial scale. The body size effect found in the second component is interesting, since *Liolaemus* species are phylogenetically related, differing mainly in the morphological variation.

Table 4. Principal component (PC) axes loadings of meristic characters for *L*. *griseus* (n = 10), *L*. *huacahuasicus* (n = 15), *L*. *orko* (n = 10), *L*. *pulcherrimus* (n = 12), and *Liolaemus* sp. nov. (n = 15). Eigenvectors, eigenvalues, and percentage of variance explained for the first five principal components from transformed data in the putative species of *Liolaemus*.

The first five components of meristic characters explained 54.76% of the variation, and a screen plot test of the PCs indicated that only those components contain relevant information. The five axes represent morphological variation, loading strongly for number of scales around midbody, number of dorsal scales between the occiput and the level of the anterior edge of the thigh, number of scales between canthal and nasal scales, subdigital lamellae of the second finger of the forelimb, and the subdigital lamellae of the third, fourth and fifth toe of the hind limb. The five axes account for the remaining variation, albeit with values below 0.70 for Hellmich index, number of lorilabials, subdigital lamellae of the first, third, fourth and fifth finger of the forelimb, subdigital lamellae of the second toe of the hind limb, number of infralabials on the left side, and the right side.

**Fig 2:**
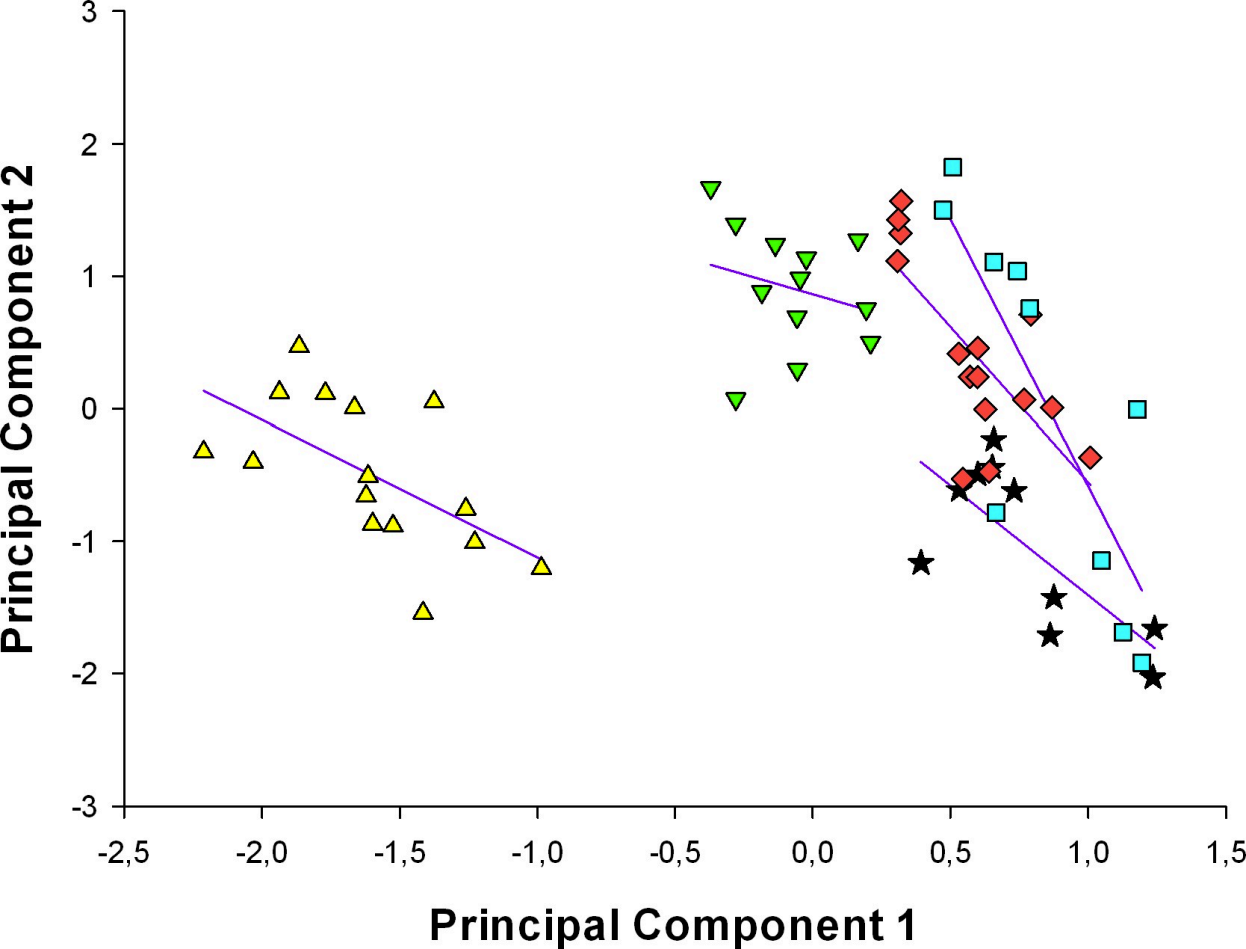
Plot of principal components scores to continuous characters for *L*. *griseus* (black stars, n = 10), *L*. *huacahuasicus* (red rhombus, n = 15), *L*. *orko* (turquoise square, n = 10), *L*. *pulcherrimus* (green triangle, n = 12), and *Liolaemus* sp. nov. (yellow triangle, n = 15). Eigenvectors, eigenvalues, and percent variation explained for the first two principal components are summarized in Table 3.

**Fig 3:**
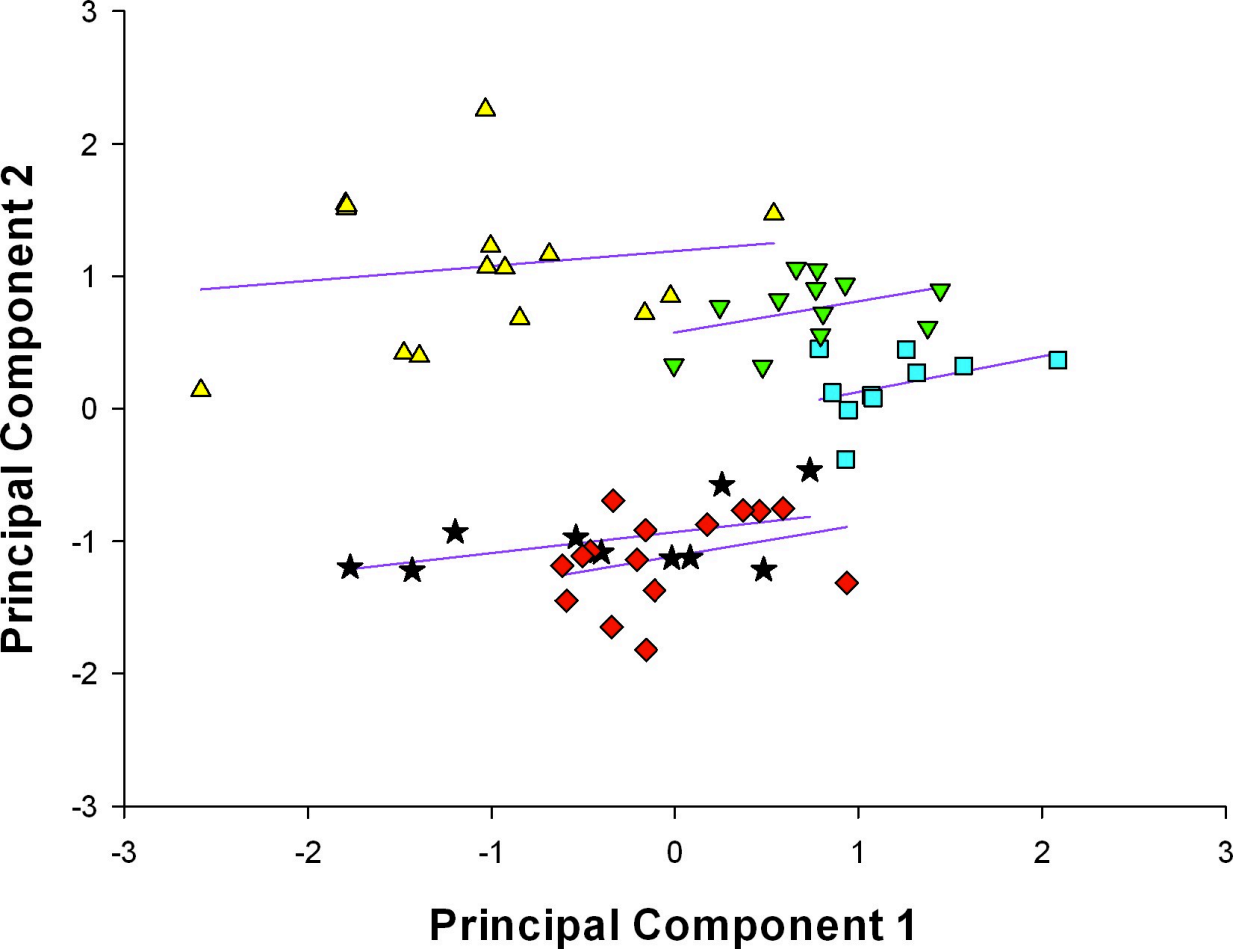
Plot of principal components score to meristic characters for *L*. *griseus* (black stars, n = 10), *L*. *huacahuasicus* (red rhombus, n = 15), *L*. *orko* (turquoise square, n = 10), *L*. *pulcherrimus* (green triangle, n = 12), and *Liolaemus* sp. nov. (yellow triangle, n = 15). Eigenvector, eigenvalues, and percent variation explained for the first five principal components are summarized in Table 4.

The position of species based on their scores of the two morphological principal components axes is illustrated in Figs 2 and 3. The spatial distribution of the continuous characters indicates that morphological variation (PC 1) and body size (PC 2) are sufficient to virtually separate the five *Liolaemus* species. These species can also be distinguished by their position analyzing meristic characters only. In both analyses, *Liolaemus* sp. nov., can be differentiated from other phylogenetically related species by its morphological variation and body size.

To further clarify the position of the *Liolaemus* species in the morphospace of both continuous and meristic characters, a DFA was carried out where the group membership was determined *a priori*. The result obtained through the DFA for the five species of *Liolaemus* was significant for continuous morphological characters (Wilk’s Lambda = 0.62, F = 3.90, P = 0.01) and the jackknife classification was 100% satisfactory. The DFA of operational taxonomic units for meristic characters was not significant (Wilk’s Lambda = 0.77, F = 1.87, P = 0.15) however; the jackknife satisfactory classification was developed at 100% rate. These results show that our *Liolaemus* sp. nov., can be reliably distinguished from the other species by a combination of morphological characters.

The results obtained by the phylogenetic and multivariate analyses with related species support the hypothesis that the population in the Tajzara Basin of the Tarija Department of the Plurinational State of Bolivia corresponds to a new species for the *L*. *montanus* group. Below we describe the new species with the formal diagnosis.

***Liolaemus tajzara*** Aguilar-Kirigin **sp. nov.** urn:lsid:zoobank.org:author:3528E2D9-DB5E-4314-8672-F9751BB52FFC

*Liolaemus islugensis* [32]

*Liolaemus islugensis* [34]

*Liolaemus islugensis* [2]

*Liolaemus* aff. *signifer* [6]

*Liolaemus* sp. 2 “Sama” [66]

*Liolaemus* sp. 2 “Torohuaico” [66]

### Holotype

**Fig 4:**
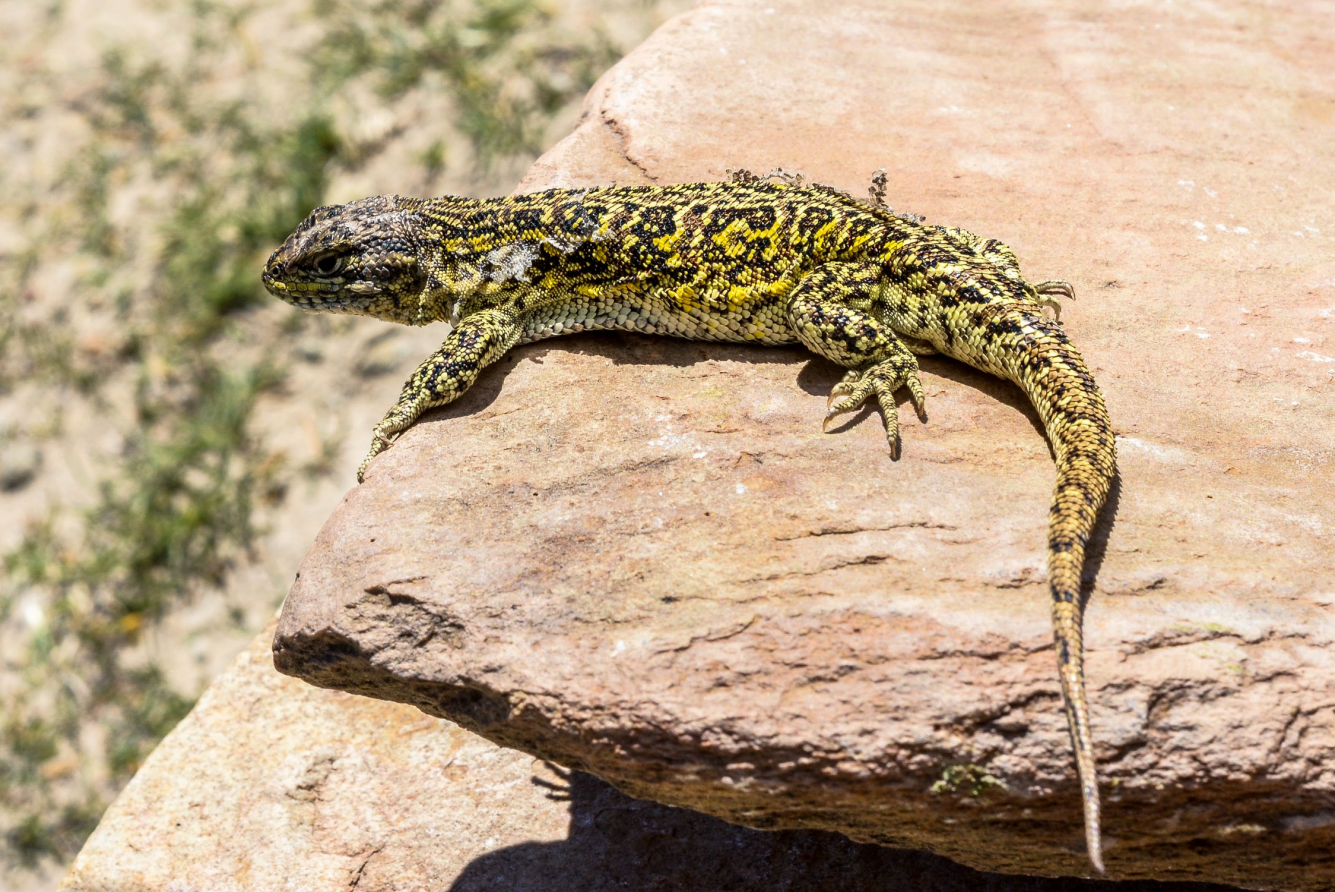
Holotype of *Liolaemus tajzara* sp. nov., dorsolateral view. Photograph: C.S. Abdala.

**Fig 5:**
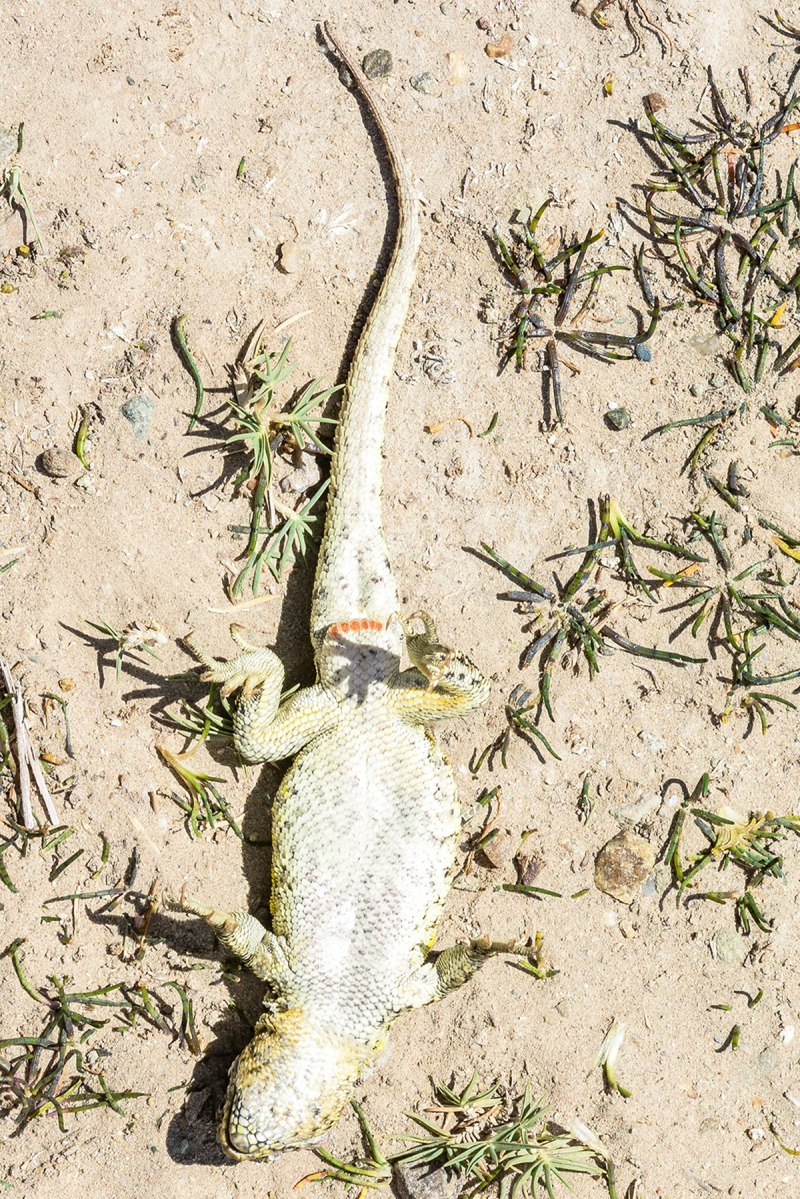
Holotype of *Liolaemus tajzara* sp. nov., ventral view. Photograph: C.S. Abdala.

CBF 4610. Adult male. Surroundings of Laguna Pujzara (21º42’21.8”S, 65º3’53.3”W), 3658 m above sea level, Reserva Biológica Cordillera de Sama, Copacabana Canton, Yunchara Municipality, Avilez Province, Tarija Department, Plurinational State of Bolivia. March 20, 2017. Collectors: Alvaro J. Aguilar-Kirigin, Cristian S. Abdala, Ana Lucia Bulacios Arroyo, and Julián Valdez (Figs 4 and 5).

### Paratypes

**Fig 6:**
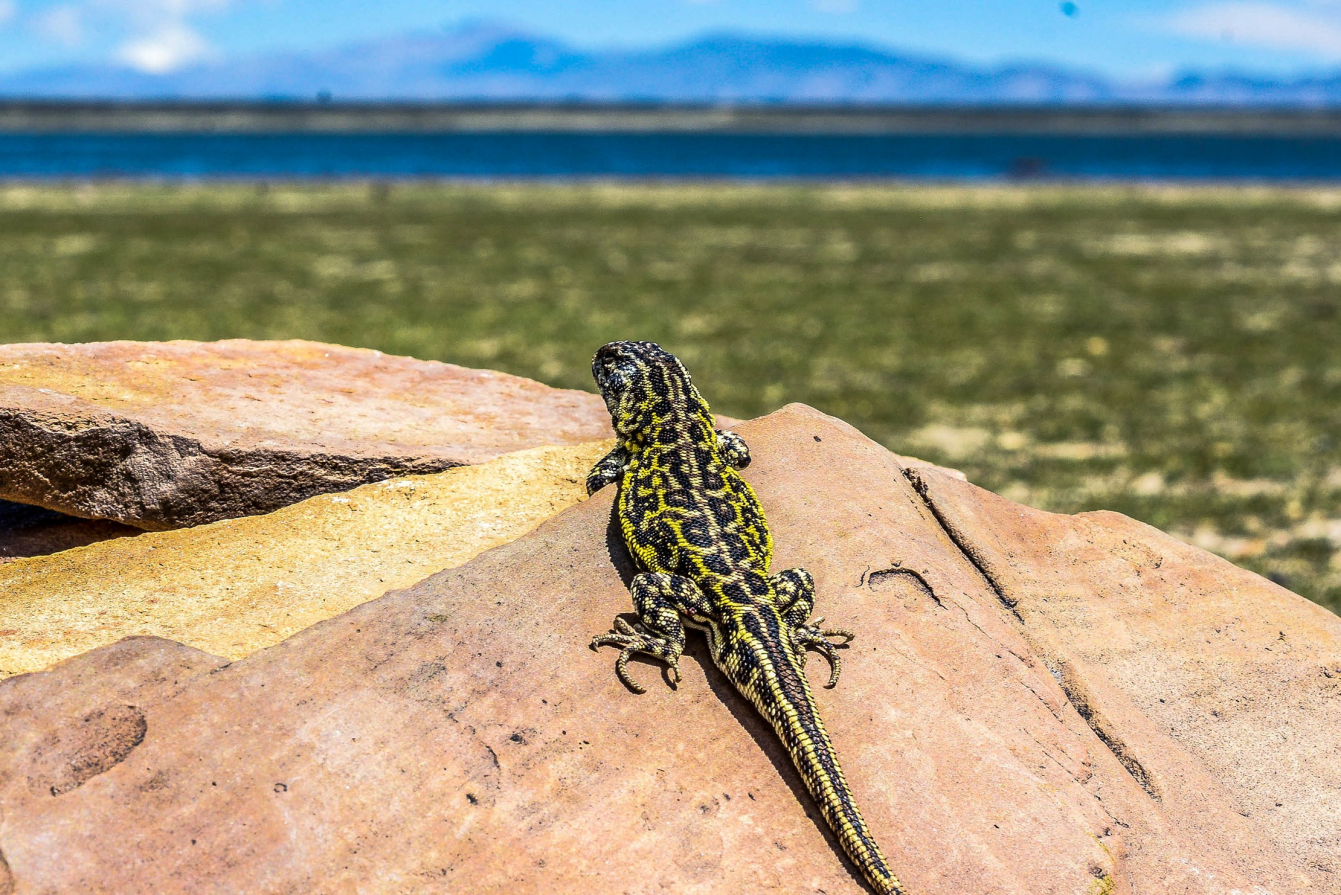
Male specimen of *Liolaemus tajzara* sp. nov., from the type locality. Photograph: C.S. Abdala.

**Fig 7:**
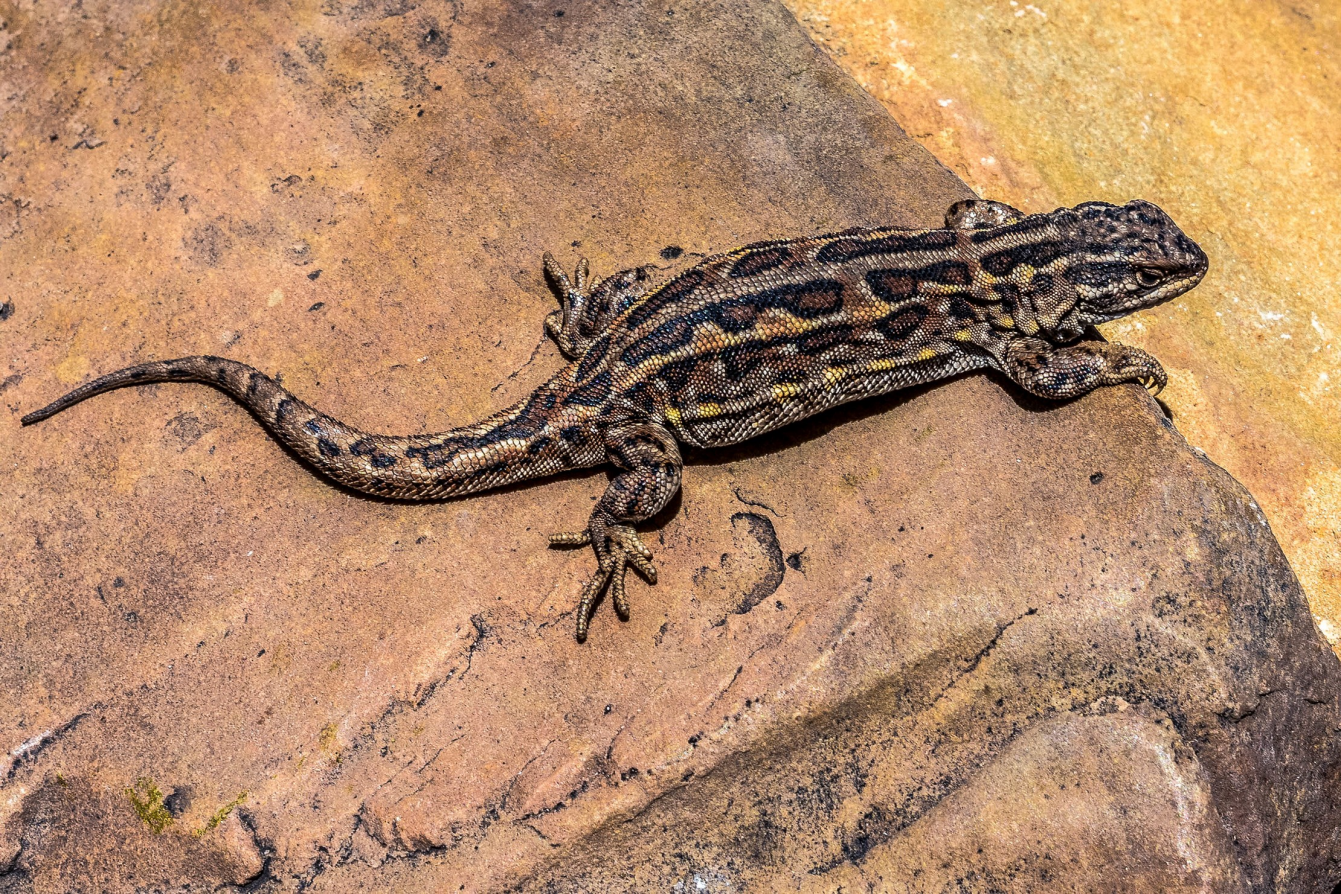
Female specimen of *Liolaemus tajzara* sp. nov., from the type locality. Photograph: C.S. Abdala.

CBF 4608–4609; 4611–4617; 4641–4643. Seven males and five females. Same data as holotype (Figs 6 and 7).

FML 3587 (1–4). Two males and two females. Surroundings of Laguna Grande and Laguna Pujzara, plains of Tajzara Basin, Reserva Biológica Cordillera de Sama (21º43’57.6”S, 65º3’36.9”W) 3691 m above sea level, Copacabana Canton, Yunchara Municipality, Avilez Province, Tarija Department, Plurinational State of Bolivia. August 09, 1995. Collectors: Gustavo Scrocchi and Pedro Blendinger.

FML 3584. Male. Río Vicuñayos, fork in the road to Vicuñayos and Arenales, Reserva Biológica Cordillera de Sama (21º45’37.3”S, 65º3’6.7”W), 3750 m above sea level, Copacabana Canton, Yunchara Municipality, Avilez Province, Tarija Department, Plurinational State of Bolivia. August 1995. Collectors: Gustavo Scrocchi and Pedro Blendinger.

FML 3581. Male. Surroundings of Laguna Grande and Laguna Pujzara, plains of Tajzara Basin, Reserva Biológica Cordillera de Sama (21º48’1.6”S, 65º2’16.5”W), 3750 m above sea level, Copacabana Canton, Yunchara Municipality, Avilez Province, Tarija Department, Plurinational State of Bolivia. August 08, 1995. Collectors: Gustavo Scrocchi and Pedro Blendinger.

### Etymology

The scientific name for this new species was assigned in reference to the type locality: the surroundings of the Tajzara Basin lagoons in the Reserva Biológica Cordillera de Sama of the Tarija Department, Plurinational State of Bolivia.

### Diagnosis

*Liolaemus tajzara* sp. nov. belongs to the *L*. *montanus* group because it presents a bladelike process on the tibia, associated with the hypertrophy of the *tibialis anticus* muscle [20, 67]. The *L*. *montanus* group species differ from those of the *L*. *boulengeri* group [9, 68] by the complete absence of patches of enlarged scales in the posterior part of the thigh (versus presence of such patches, at least in adult males). Within the *L*. *montanus* group, our new species is distinguished from *Liolaemus audituvelatus*, *L*. *famatinae*, *L*. *griseus*, *Liolaemus insolitus*, *Liolaemus manueli*, *Liolaemus omorfi*, *Liolaemus poconchilensis*, *Liolaemus reichei*, *Liolaemus stolzmanni* and *Liolaemus torresi* by their smaller size, with a maximum SVL of less than 65 mm compared to at least 71.9 mm in *L*. *tajzara* sp. nov. and, it is distinguished from *Liolaemus annectens*, *Liolaemus aymararum*, *L*. *chlorostictus*, *L*. *dorbignyi*, *Liolaemus duellmani*, *Liolaemus fabiani*, *Liolaemus filiorum*, *L*. *forsteri*, *Liolaemus foxi*, *Liolaemus huayra*, *L*. *huacahuasicus*, *Liolaemus igneus*, *Liolaemus inti*, *L*. *jamesi*, *Liolaemus juanortizi*, *Liolaemus melanogaster*, *Liolaemus nigriceps*, *L*. *orientalis*, *L*. *pantherinus*, *L*. *pachecoi*, *Liolaemus patriciaiturrae*, *L*. *pleopholis*, *Liolaemus polystictus*, *L*. *puritamensis*, *Liolaemus robustus*, *Liolaemus scrocchii*, *L*. *signifer*, *Liolaemus tacora*, *Liolaemus vulcanus* and, *Liolaemus williamsi* by their larger size with maximum SVL exceeding 75 mm (often greater than 90 mm) versus the observed maximum of 71.9 mm in *L*. *tajzara* sp. nov.

**Fig 8:**
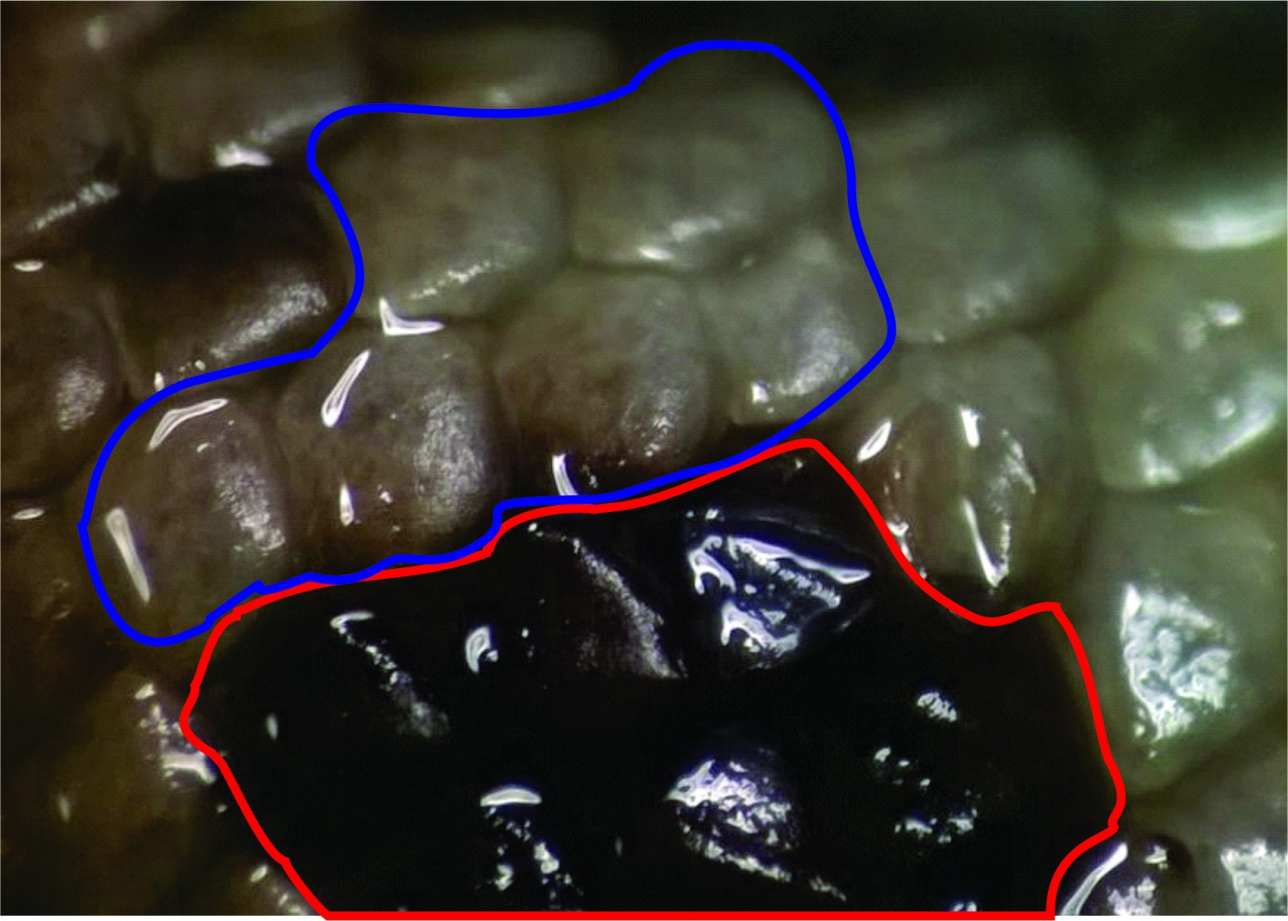
Scales of the back of the body of *L*. *tajzara* sp. nov. exhibiting keeled dark scales and smooth in the light-colored scales.

*Liolaemus tajzara* sp. nov. has a unique character among the analyzed *Liolaemus* of the *L*. *montanus* group, and that is that the keels of the dark dorsal scales are more evident than those of the light scales, which can even be smooth (Fig 8). The presence of sub-imbricate dorsal scales, with slight keels, differentiates *L*. *tajzara* sp. nov. from species with smooth juxtaposed or sub-imbricate scales such as *Liolaemus andinus*, *L*. *audituvelatus*, *Liolaemus cazianiae*, *Liolaemus eleodori*, *L*. *erguetae*, *L*. *fabiani*, *L*. *foxi*, *Liolaemus gracielae*, *Liolaemus halonastes*, *L*. *insolitus*, *L*. *islugensis*, *L*. *jamesi*, *Liolaemus molinai*, *L*. *manueli*, *L*. *nigriceps*, *L*. *omorfi*, *L*. *patriciaiturrae*, *L*. *pleopholis*, *L*. *poconchilensis*, *Liolaemus poecilochromus*, *Liolaemus porosus*, *L*. *reichei*, *Liolaemus robertoi*, *L*. *robustus*, *Liolaemus rosenmanni*, *Liolaemus ruibali*, *L*. *schmidti*, *L*. *scrocchii*, *L*. *tacora*, *L*. *torresi*, *Liolaemus vallecurensis* and *L*. *vulcanus*. Also, *L*. *tajzara* sp. nov. differs from species with imbricate dorsal scales with evident keels, such as *L*. *annectens*, *L*. *aymararum*, *Liolaemus disjunctus*, *L*. *etheridgei*, *L*. *fittkaui*, *L*. *griseus*, *L*. *huacahuasicus*, *L*. *montanus*, *L*. *orientalis*, *L orko*, *L*. *pachecoi*, *L*. *pulcherrimus*, *L*. *signifer*, *Liolaemus thomasi* and *L*. *williamsi*. This new species differs from *L*. *evaristoi* and *L*. *etheridgei* by the absence of sky blue or celeste scales on the sides and dorsum of the body and tail.

**Fig 9:**
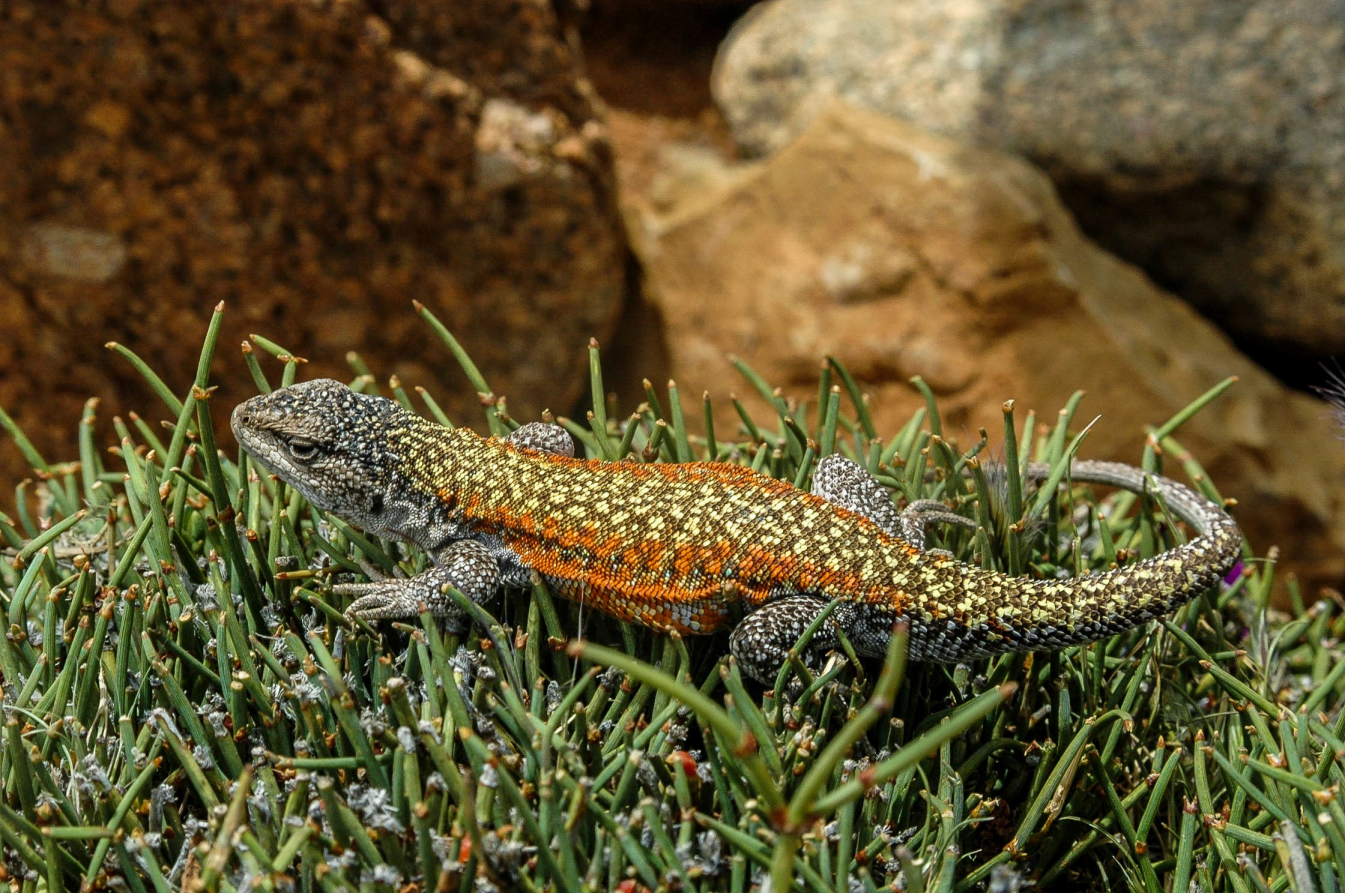
Male specimen of *L*. *pulcherrimus* from Mudana, Jujuy, Argentina, dorsolateral view. Photograph: C. S. Abdala.

**Fig 10:**
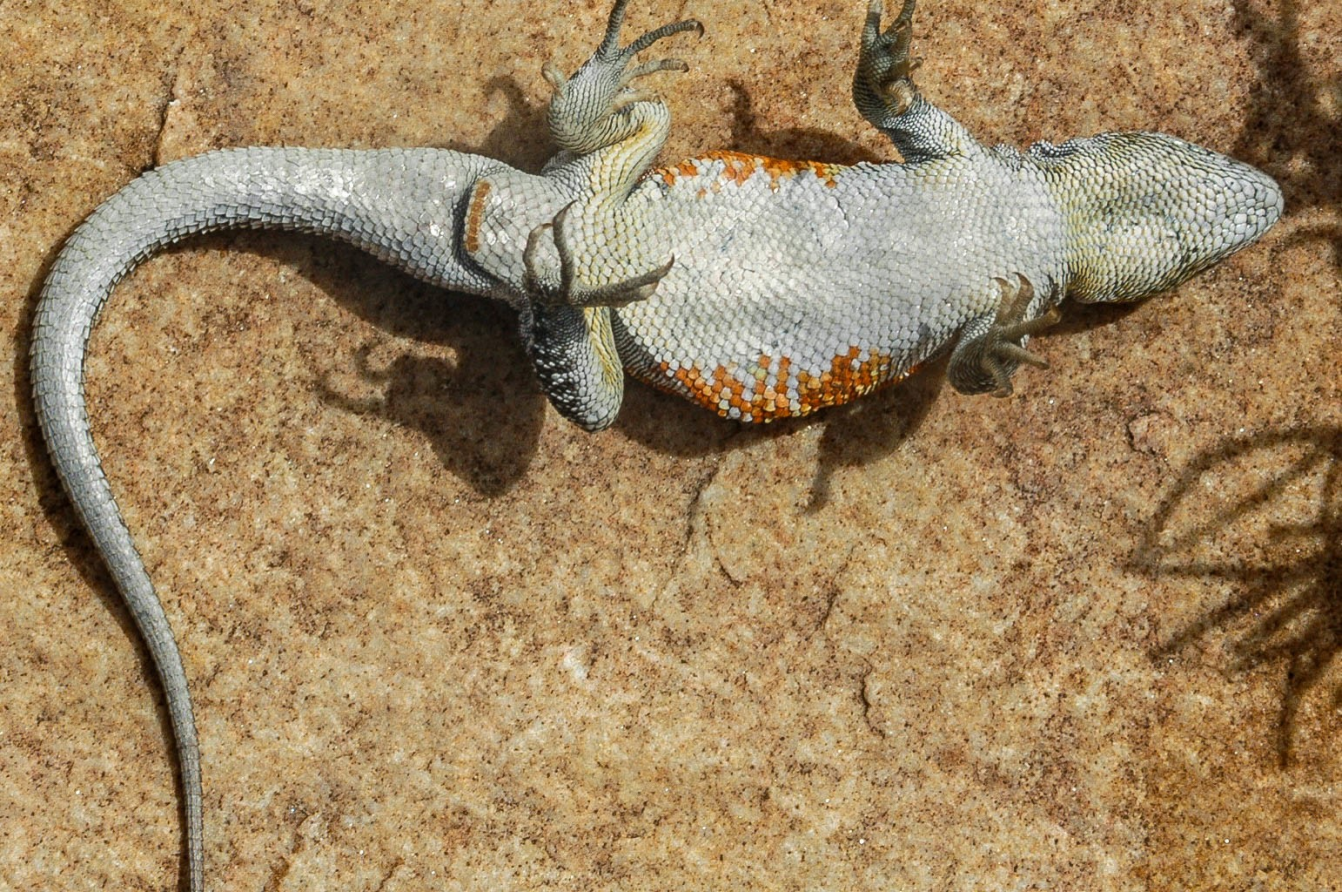
Male specimen of *L*. *pulcherrimus* from Mudana, Jujuy, Argentina, ventral view. Photograph: C. S. Abdala.

The number of scales around midbody in *L*. *tajzara* sp. nov. varies between 66 and 82 (mean = 72.2), which differentiates it from several species of the group with more than 85 scales, such as *L*. *andinus*, *L*. *eleodori*, *L*. *erguetae*, *L*. *gracielae*, *L*. *halonastes*, *L*. *molinai*, *L*. *nigriceps*, *L*. *patriciaiturrae*, *L*. *pleopholis*, *L*. *porosus*, *L*. *robertoi*, *L*. *rosenmannii*, and *L*. *vallecurensis* and from species with less than 66 such as *L*. *annectens*, *L*. *aymararum*, *L*. *chlorostictus*, *L*. *dorbignyi*, *L*. *etheridgei*, *L*. *fabiani*, *L*. *famatinae*, *L*. *fittkaui*, *L*. *griseus*, *Liolaemus hajeki*, *L*. *huayra*, *L*. *huacahuasicus*, *L*. *jamesi*, *L*. *igneus*, *L*. *melanogaster*, *L*. *montanus*, *L*. *orientalis*, *Liolaemus ortizi*, *L*. *pachecoi*, *L*. *poconchilensis*, *L*. *puritamensis*, *L*. *thomasi*, *L*. *robustus*, *L*. *vulcanus* and *L*. *williamsi*. The number of ventral scales between the mental scale and the border of the vent in *L*. *tajzara* sp. nov. varies between 75 and 90 (mean = 80.9), and is lower than in the following species, with more than 90 ventral scales: *L*. *andinus*, *L*. *cazianiae*, *L*. *erguetae*, *L*. *foxi*, *L*. *gracielae*, *L*. *halonastes*, *L*. *inti*, *Liolaemus multicolor*, *L*. *nigriceps*, *L*. *pachecoi*, *L*. *patriciaiturrae*, *L*. *pleopholis*, *L*. *poecilochromus*, *L*. *porosus*, *L*. *robertoi*, *L*. *rosenmannii*, *L*. *torresi*, and *L*. *vallecurensis*; and higher than in the following species with less than 75 ventral scales: *L*. *dorbignyi*, *L*. *fittkaui*, *L*. *melanogaster*, *L*. *ortizi*, *L*. *polystictus*, *L*. *robustus*, and *L*. *thomasi*. Likewise, the dorsal patterns of most males and females, formed by ocelli-like paravertebral spots with black borders and light brown centers, and by evident continuous or discontinuous dorsolateral bands, clearly differentiate it from its closest relatives (*L*. *fittkaui*, *L*. *griseus*, *L*. *huacahuasicus*, *L*. *montanus*, *L*. *orko* and *L*. *pulcherrimus*) [6] and from the rest of the *Liolaemus*, while being most similar in this respect to some female *L*. *fittkaui*. However, *L*. *tajzara* sp. nov. is phylogenetically closest to *L*. *pulcherrimus* (Fig 1, Figs 9 and 10) [6]. In addition to the differences highlighted above, they differentiate from each other in that *L*. *tajzara* sp. nov. presents a lower number of dorsal scales from the occiput to the hind limbs (69–94, mean = 73.8 vs. 78–90, mean = 85.3) and that females present 0 to 4 precloacal pores versus up to 5 in *L*. *pulcherrimus*.

### Description of holotype

Adult male (CBF 4610). SVL 71.40 mm. Head 1.17 times longer (15.34 mm) than wide (13.13 mm). Head height 9.03 mm. Neck width 8.89 mm. Eye diameter 3.48 mm. Interorbital distance 7.79 mm. Orbit-auditory meatus distance 6.66 mm. Auditory meatus 2.62 mm high, 1.48 mm wide. Orbit-commissure of mouth distance 2.61 mm. Internasal width 2.79 mm. Subocular scale 4.02 mm. Trunk length 32.74 mm, width 21.98 mm. Tail length 78.12 mm. Femur length 12.74 mm, tibia 13.36 mm, and foot 17.98 mm. Humerus length 8.20 mm. Forearm length 8.58 mm. Hand length 10.51 mm. Pygal region length 8.72 mm, and cloacal region width 9.52 mm (Figs 4 and 5).

Dorsal surface of the head smooth, with sixteen scales, rostral wider than tall, bordered by six scales. Mental larger than rostral, trapezoidal, bordered by four scales. Nasal not in contact with rostral. Two internasals longer than wide. Nasal surrounded by eight scales, separated from canthal by two scales. Seven scales between frontal and rostral. Frontal divided. Two postrostrals. Interparietal smaller than parietals, in contact with seven scales. Preocular separated from lorilabial row by one scale. Seven superciliaries and fifteen upper ciliaries. Differentiated scales at anterior margin of auditory meatus. Twelve smooth temporals. Four lorilabials in contact with subocular. Seven supralabials, not in contact with subocular. Five supraoculars. Eight lorilabials. Five infralabials. Five chin shields, second pair separated by two scales. Seventy-one scales around midbody. Seventy-eight rounded, juxtaposed and slightly keeled dorsal scales between occiput and hind limbs. Forty scale transverse rows in dorsum. Eighty ventral scales from mental to the cloacal region, following the ventral midline of the body, larger than dorsal scales, flat, imbricate. Twenty-five smooth, imbricate gulars. Thirty-seven scales longitudinal fold of the neck. Six precloacal pores. Antehumeral scales larger and easily distinguishable from the rest. Auricular and longitudinal folds present. Scales on the longitudinal fold granular and smooth. Fourth finger with eighteen subdigital lamellae; fourth toe with twenty-four subdigital lamellae. Infracarpals and infratarsals with smooth and imbricate scales. Gular fold present. Dorsal tail scales with mucron and keel, ventral tail smooth. Scales from posterior thigh rounded posteriorly without notch. Scales without keel in the center of the palm of the foot.

### Color in life

Head light brown, with distinguishable black spots in the supraocular and occipital region. Three dark spots in the temporal region parallel with each other and to the head axis, delimited by thin white stripes. The two lower stripes more evident and continue to the sides of the neck. Supralabial and loreolabial scales yellow, infralabial scales lighter, with black border. Body light brown with several scales and irregular light yellow spots, and dim. Two rows of paravertebral irregular spots in the form of irregular ocelli with black border and brown interior, with few scattered yellow scales, distinguished on the dorsum. Such pairs of ocelli extend throughout the length of the tail, where they merge in a unique central band from the first third. Small spots and black scales irregularly distributed on the vertebral region. Without vertebral line. With dorsolateral stripes, fragmented and discontinuous of an intense yellow. With lateral spots on the flanks of the body, of the same color and irregular form than paravertebral spots. Intense yellow spots distinguished between the lateral spots. Without scapular spots. Forelimbs and hind limbs dorsally light brown, with several spots and yellow, dark scales arranged irregularly. Fingers and toes cream-colored. Tail dorsally of same color and design as the rest of the body. Without blue scales on the body and the tail. Mental region ventrally whitish with interstices between the gray scales and scattered yellow spots. Gular region white with yellow sides. Pectoral region, abdominal region, pygal region, and ventral surfaces of tail and limbs white with certain scales or dark spots shaped like specks on the sides of the gulars, on the pygal region, and on the tail. Precloacal pores of an intense orange.

### Variation

Based on twelve specimens (seven males and five females). Dorsal surface of the head smooth with 16–21 scales (mean = 17.42; SD = 1.51). Nasal surrounded by 5–8 (mean = 6.50; SD = 1.17) scales. Supralabials 6–8 (mean = 7.25; SD = 0.87), lorilabials 7–9 (mean = 8.33; SD = 0.78). One row of lorilabials. Supraoculars 5–7 (mean = 5.83; SD = 0.72). Interparietal always smaller than parietals, surrounded by 4–9 (mean = 6.75; SD = 1.36) scales. Infralabials 5–6 (mean = 5.42; SD = 0.51). Gulars 23–28 (mean = 25.5; SD = 1.78). Temporals 11–13 (mean = 12.42; SD = 0.79) and smooth. Auditory meatus higher 1.55 to 2.90 mm (mean = 2.41 mm; SD = 0.41) than wide 1.07 to 1.76 mm (mean = 1.39 mm; SD = 0.24). Head longer 12.52 to 15.69 mm (mean = 14.28 mm; SD = 1.29) than wide 10.49 to 13.33 mm (mean = 11.96 mm; SD = 1.06). Head height 7.10 to 9.97 mm (mean = 8.31 mm; SD = 0.86). Trunk length 26.54 to 33.17 mm (mean = 30.21 mm; SD = 2.45). Males SVL 63.60 to 71.87 mm (mean = 67.89 mm; SD = 3.07) and females 60.44 to 66.71 mm (mean = 63.24 mm; SD = 2.77). Femur length 8.64 to 13.40 mm (mean = 11.36 mm; SD = 1.56). Humerus length 6.36 to 8.29 mm (mean = 7.38 mm; SD = 0.73). Forearm length 6.29 to 8.60 mm (mean = 7.70 mm; SD = 0.64). Hand length 8.95 to 12.11 mm (mean = 10.24 mm; SD = 0.85). Scales around midbody 66–82 (mean = 72.17; SD = 4.28). Dorsals 69–94 (mean = 77.83; SD = 6.01) juxtaposed and slightly keeled between occiput and limbs. Infradigital lamellae on fourth finger 15–19 (mean = 16.00; SD = 1.21) and 20–24 (mean = 21.50; SD = 1.17) on fourth toe. Infracarpals and infratarsals with smooth and imbricate scales. Ventral scales 75–90 (mean = 80.92; SD = 4.46) larger than dorsal scales 69–94 (mean = 77.83; SD = 6.01). Tail length 53.36 to 69.41 mm (n = 7; mean = 63.96 mm; SD = 6.00). Males with 6–9 (mean = 7.71; SD = 1.25) precloacal pores and females with 0–4 (mean = 0.80; SD = 1.79) precloacal pores.

### Color in life

*Liolaemus tajzara* sp. nov. shows evident sexual dichromatism (Figs 4–7). In males head varies dorsally from light to dark brown, always with black spots. The central spot is always widest and can contain a brown spot on its center, while the superior spot is generally more marked than the inferior spot. Three bold black stripes on the sides of the head and temporal region, parallel to each other, delimited by thin white stripes. These stripes run from the posterior part of the eye towards the neck, with the two upper stripes integrating to the patterns of the side of the neck. The inferior stripe reaches the auditory meatus. Loreolabial, supralabial and infralabial scales generally yellow or white, always lighter than the rest of the head. In few specimens the infralabial scales present black borders, and the rest of the scale is light-colored. No scapular spots or ante humeral arch observed. Neck dorsally has the same color and pattern design as the rest of the body. Two series of prominent paravertebral spots in the form of irregular ocelli, with brown center and intense black border (Fig 6). These ocelli vary in size, shape and color intensity according to the specimen. Over these ocelli some yellow scales can protrude. These series of ocelli occasionally merge longitudinally but never across the vertebral region. This region is well delimited, without vertebral line and with small specks and black scales irregularly distributed. In most of the observed males, thin light lines, sometimes fragmented, surround the dark ocelli. In some specimens, thin dorsolateral yellow lines are formed, while in others they can be fragmented or present only in the form of irregular spots. On the sides of the body, lateral spots in the form of ocelli protrude, of the same color and irregular shape than the paravertebral spots. Scales and white, black and yellow spots irregularly scattered between the lateral spots. Lateral midline of the body generally yellow, with small dark and light spots that do not form a regular design below it. Fore and hind limbs of the same color as the dorsum of the body, generally with many dark spots. Tail with the same coloration pattern and design as the dorsum of the body, with paravertebral spots merging on the base, forming a single spot on the vertebral region. Single longitudinal spot with black borders and light brown center along the tail in certain specimens. Most males ventrally white, with dark mole-shaped spots. Yellow color in the mental and gular region in certain specimens, and light orange color in the tail and hind limbs in one specimen.

Females generally with the same spot design and pattern than males, but with other combinations of colors (Fig 7). Striking form and design of paravertebral spots, but with less pronounced coloration. When present, yellow or orange scales on the dorsum and sides of the body, almost indistinguishable at first sight. Paravertebral and lateral ocelli reddish brown in the center, with black borders. Ventral color white, with scattered darker spots on the abdomen in some specimens.

## Distribution

**Fig 11:**
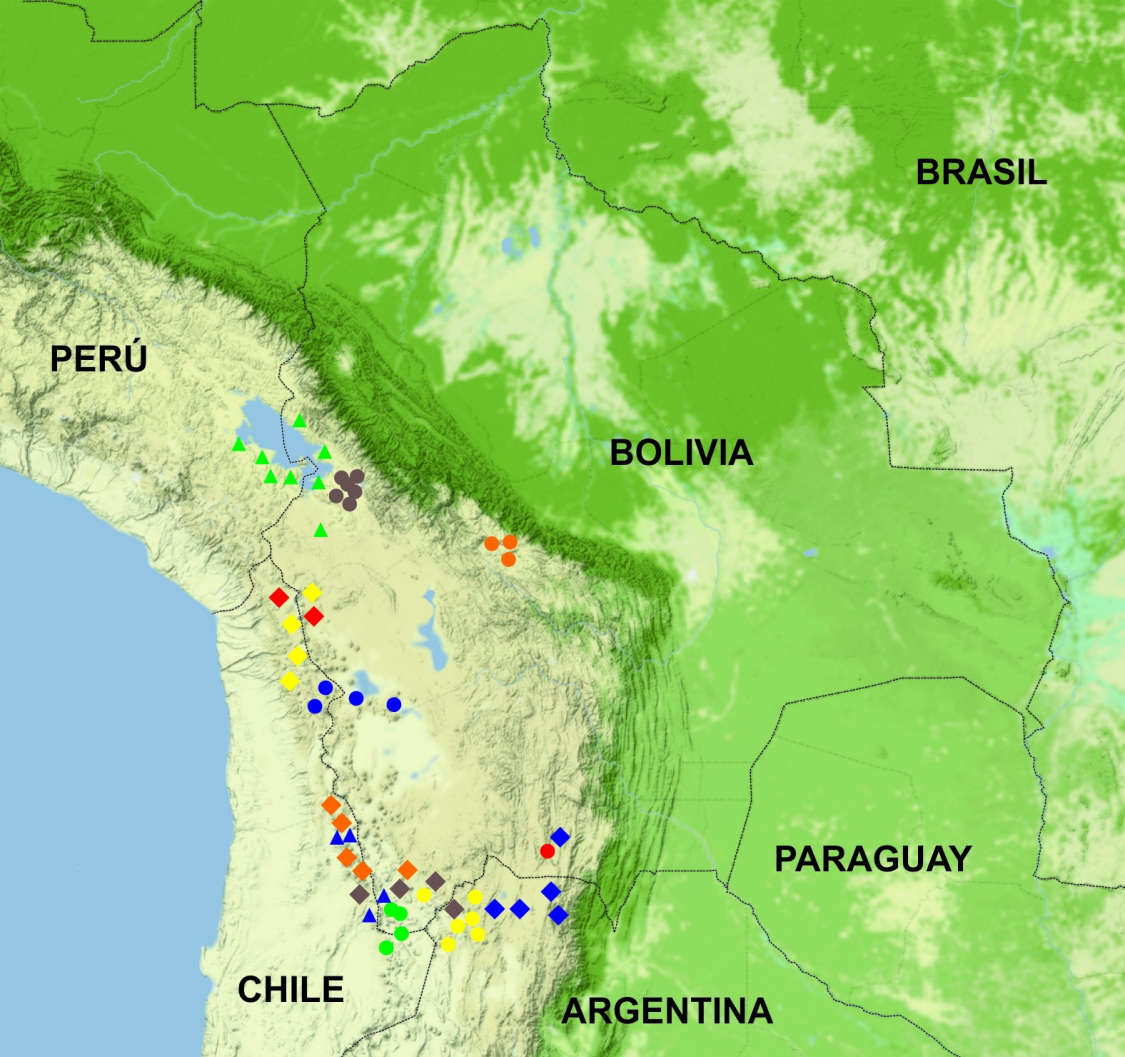
**Bolivia map showing the distribution of species of the *L*. *montanus* group:** (A) *L*. *tajzara* sp. nov.: red circle (21º42’21.8”S 65º3’53.3”W, 21º43’57.6”S 65º3’36.9”W, 21º45’37.3”S 65º3’6.7”W, 21º48’1.6”S 65º2’16.5”W). (B) *L*. *chlorostictus*: yellow circle (22º16’42.9”S 67º3’21.6”W). (C) *L*. *erguetae*: green circle (21º44’23.7”S 67º23’48.5”W, 22º12’33.1”S 67º42’29.6”W). (D) *L*. *fittkaui*: orange circle (17º19’34.0”S 65º50’3.4”W, 17º22’43.3”S 65º39’51.8”W, 17º28’4.9”S 65º33’40.1”W). (E) *L*. *forsteri*: brown circle (16º18’49.7”S 68º9’27.0”W, 16º20’56.5”S 68º1’44.7”W, 16º22’35.4”S 68º5’26.7”W, 16º22’2.5”S 68º8’45.1”W, 16º30’45.4”S 67º59’45”W, 16º38’40.9”S 67º50’14.6”W). (F) *L*. *islugensis*: blue circle (18º57’48.4”S 66º42’52.2”W, 19º2’46.1”S 68º3’28.6”W). (G) *L*. *jamesi*: yellow diamond (18º9’0”S 69º7’0’W). (H) *L*. *orientalis*: blue diamond (21º40’59.5”S 65º0’31.6”W). (I) *L*. *pachecoi*: orange diamond (22º12’33.1”S 67º42’29.6”W). (J) *L*. *pleopholis*: red diamond (18º7’0”S 69º2’0”W, 18º8’11.8”S 68º58’31.5”W, 18º10’7.4”S 68º43’18.7”W, 18º19’31.7”S 68º53’18”W). (K) *L*. *puritamensis*: brown diamond (22º11’16”S 67º20’18.3”W, 22º16’42.9”S 67º3’21.6”W, 22º27’51.6”S 67º35’42.1”W). (L) *L*. *schmidti*: blue triangle (22º12’33.1”S 67º42’29.6”W). (M) *L*. *signifer*: green triangle (15º37’39.6”S 69º1’52.5”W, 16º11’56.8”S 68º28’12.2”W, 16º32’53.4”S 68º40’46.1”W, 17º13’9.1”S 67º52’57.2”W).

**Fig 12:**
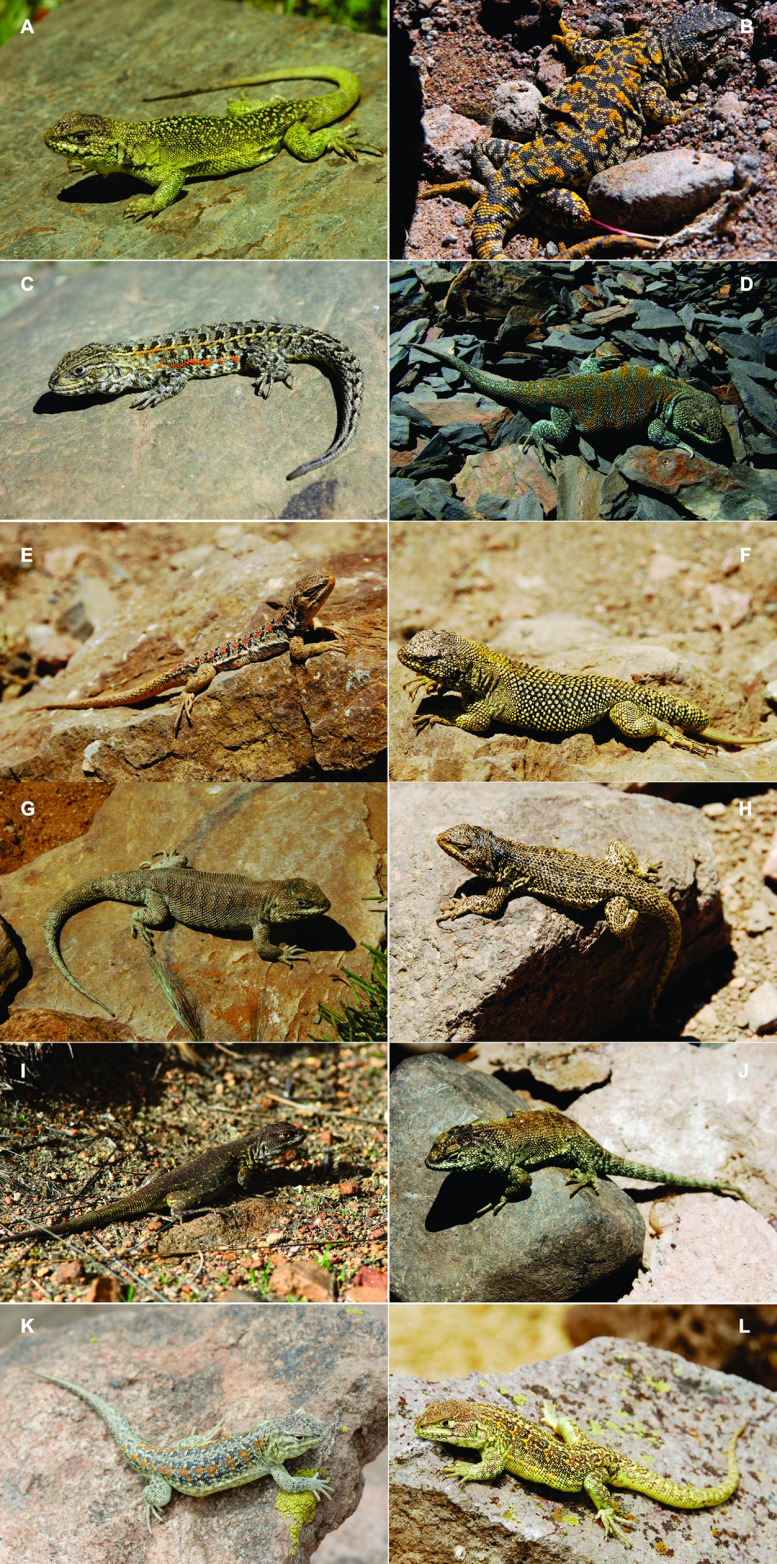
Plate with photos of male specimens of the different species that we consider valid of *L*. *montanus* group from Bolivia. (A) *L*. *chlorostictus*. (B) *L*. *erguetae*. (C) *L*. *fittkaui*. (D) *L*. *forsteri*. (E) *L*. *islugensis*. (F) *L*. *jamesi*. (G) *L*. *orientalis*. (H) *L*. *pachecoi*. (I) *L*. *pleopholis*. (J) *L*. *puritamensis*. (K) *L*. *schmidti*. (L) *L*. *signifer*.

All known specimens and observations of *L*. *tajzara* sp. nov. are from the Reserva Biológica Cordillera de Sama, Yunchara Municipality, Avilez Province, Tarija Department, Plurinational State of Bolivia, mainly in the semi-humid Puna phytogeographic region, at altitudes higher than 3500 m (Figs 11 and 12).

### Natural history and conservation status

*Liolaemus tajzara* sp. nov. is an endemic lizard of Tajzara Basin, where altitudes range from 3600 to 4700 m. This ecoregion is characterized by a cold and arid climate with strong winds and scarce precipitation, including rain and hail; minimum temperature in dry season reaches –18°C and maximum temperature in wet season reaches 22°C. Vegetation is composed mainly by bunchgrasses (*Festuca orthophylla*, *Festuca chrysophylla*, *Stipa leptostachya*), “tola” shrubs (*Baccharis incarum*, *Baccharis boliviensis*), “kanllar” (*Tetraglochin cristatum*), “yareta” cushion plants (*Azorella compacta*) and patches of queñoa forests (*Polylepis tomentella*). In the basin there are several saline lagoons, both temporal and permanent, inhabited by flamingos (*Phoenicoparrus andinus*, *Phoenicoparrus jamesi*, *Phoenicopterus chilensis*), many duck species (Anatidae spp.), Andean geese (*Chloephaga melanoptera*), horned coots (*Fulica cornuta*) and other aquatic bird species. In the highlands there are condors (*Vultur gryphus*), and towards the eastern slopes there are taruca (*Hippocamelus antisensis*), which move to the lowlands during the dry season months. Other native fauna of the area include tuco-tucos (*Ctenomys lewisi*) and mice (*Akodon* spp.). *Liolaemus tajzara* sp. nov. protect themselves from the extreme weather conditions by building underground burrows. The entire known of population of *L*. *tajzara* sp. nov. is found within the Reserva Biológica Cordillera de Sama. Given that its estimated area of occupancy is much less than 2000 km^2^, that it is known from less than 10 locations, and that we believe that projected climate change for the high Andes is likely to lead to a decline in the area of suitable habitats and thus put this high-altitude viviparous species at risk [69, 70, 71], we recommend that *L*. *tajzara* sp. nov., be listed as Vulnerable B.2ab following the IUCN Red List criteria.

## Discussion

Until a few years ago, little was known about the taxonomy, systematics, and phylogenetics of the *Liolaemus* species present in the Plurinational State of Bolivia. The work by Langstroth [12] on *L*. *stolzmanni*, *L*. *reichei*, and *Liolaemus jamesi pachecoi* points out that the type material of the majority of the species of this genus described in the past is kept largely in European and North American museums, which for many decades limited herpetological research in Bolivia. However, the taxonomic revision of the “*alticolor*-*bibronii*” group in the scientific collections of the Colección Boliviana de Fauna allowed for the description of *Liolaemus aparicioi* from the dry inter-Andean valleys just below the city of La Paz [72]. Similarly, exhaust revisions of specimens housed in the major museums of Bolivia during the last few years has led to the establishment of new records for various species: *L*. *chacoensis*, Reserva Natural “El Corbalán”, Gran Chaco Province, Tarija [73], *L*. *chaltin*, Reserva Biológica Cordillera de Sama, Avilez Province, Tarija [74], *L*. *puna*, Nor Chichas Province, Potosí and in the Reserva Biológica Cordillera de Sama, at Laguna Grande, Méndez Province [75], *L*. *puritamensis*, Sur Lipez Province, Potosí [29], and *L*. *pleopholis*, Sajama Province, Oruro [76]. In addition, range extensions in Bolivia were proposed for *L*. *chacoensis* [77] and *L*. *variegatus* [35]. Finally, the revision of the range of *L*. *signifer* [78] highlights the need for a historical bibliographic review of the species, as well as a taxonomic study of the supposed rediscovered holotype, the populations around Lake Titicaca, and morphologically similar taxa such as *L*. *pleopholis*.

Despite these advances, our understanding of the Bolivian members of the *L*. *montanus* group was nearly stagnant since Pellegrin [79] until the pioneering work of Raymond Laurent from the early 1980s through the late 1990s, followed again by another period of stagnation. Prior to *L*. *tajzara* sp. nov. the last species of the *L*. *montanus* group described from Bolivia were *L*. *erguetae* and *L*. *pachecoi*, both described by Laurent in 1995 [80]. The classic morphological and morphometric approach of Laurent using informative characters lead to the identification and description of the following species from Bolivia: *L*. *erguetae* [80], *L*. *fittkaui* [81], *L*. *forsteri* [82], *L*. *pachecoi* [80], and *L*. *variegatus* [83], all of which are currently recognized as valid taxa. Following Laurent’s studies, the morphological-morphometric approach continued to develop, in particular in regards to the origins of characters, for example, the work of Aguilar-Kirigin [33] which correlated continuous characters in a PCA to delimit species as function of body size. The findings of Aguilar-Kirigin [33] were in line with the hypothesis of Laurent [84], which was subsequently confirmed by the macromolecular studies of Schulte et al. [68]. These studies have contributed to a better understanding of the species richness of a region of Bolivia, which still remains underexplored.

The description of the new *Liolaemus* species from southernmost Bolivia increases our understanding of the herpetological richness of the region, as did the morphological and taxonomic studies of Harvey and Gutberlet [85] in the isolated Huanchaca Range of the Santa Cruz Department of Bolivia, which allowed them to identify and describe *Tropidurus callathelys*, *Tropidurus chromatops*, and *Tropidurus xanthochilus* as new species. Likewise, the studies of the genus *Stenocercus* by Torres-Carvajal [86, 87] which include the descriptions and redescriptions of many new or poorly understood species. As noted by Torres-Carvajal [86], approximately a quarter of the species of the genus were described after the year 1990, now more than 180 years after the description of the type species *Stenocercus roseiventris* [88] from Bolivia. According to Torres-Carvajal [87], one of the principal causes for the discovery of the many of these new species in recent decades has been the exhaustive morphological review of material in museums, as many collections often hold undescribed material that represent species new to science. In recognition of these findings, we are faced with the urgent need to complete and support the herpetological collections in Bolivia, in agreement with the conclusions of Langstroth [12, 89], who noted that many species remained to be described from Bolivia, especially in *Liolaemus*, *Stenocercus*, and *Tropidurus* in the underexplored regions of the country. In the case of *L*. *tajzara* sp. nov., the first specimens were collected in 1995 and deposited in the Colección Boliviana de Fauna and Fundación Miguel Lillo, where they remained unstudied until our work on the Bolivian members of the *L*. *montanus* group began in 2010. These specimens are representative of the many of others in Bolivian collections which await study.

Abdala et al. [22] noted that from 1998 to 2007 an average of five new species of *Liolaemus* were described annually, but from 2008 to date, 66 new species have been described for an average of 6.5 new *Liolaemus* per year. The principal evidence used in support of these taxa has been morphological [1, 6, 15, 16, 90, 91, 92, 93, 94], molecular [14, 66, 95, 96, 97, 98, 99], morphological and molecular [5, 19, 100, 101, 102], morphological, molecular, and cytogenetic [18], and phylogenetic [2, 103]. This taxonomic research demonstrates that *Liolaemus* have morphological characters that are informative for the delimitation of species and that many of these characters are likely to be adaptive, allowing these lizards to exploit a wide range of habitats and macroenvironments, as expressed by the high species richness of the genus [104].

To evaluate our hypothesis that *L*. *tajzara* sp. nov. is a new species to science we adopt a comparative approach, evidencing that this population of lizards inhabiting the isolated Tajzara Basin in the Cordillera de Sama of the Tarija Department can be distinguished by morphological characters, which we have supported with multivariate analyses of continuous and meristic characters. Given that species are interrelated through ancestor-descendent lineages, species can share similar character states that have been inherited from a common ancestor (i.e., plesiomorphic characters), which means that characters shared by species may not be independent; as such, we analyze the relationships among species through a phylogenetic perspective.

Given that our hypothesis is supported by our morphological analyses, we consider the molecular studies of Aguilar-Puntriano et al. [66] and the thermal ecology studies of Jiménez-Robles and De la Riva [105] to provide independent evidence that supports our hypothesis. In their research on convergence within the *L*. *montanus* group, Aguilar-Puntriano et al. [66] recovered a lineage they identified as “sp2 Sama” and “sp2 Torohuaico” (from the Tajzara Basin) based on molecular evidence. Their phylogenetic estimate includes specimens representing the populations we describe here as *L*. *tajzara* sp. nov., which they identify as *Liolaemus* sp. 2 in the following phylogenetic position: (*L*. sp. 2 + ((*L*. *islugensis* + *L*. *pleopholis*) (*L*. *orientalis* (*L*. sp. 1 (*L*. *multicolor* (*L*. sp. 4 + *L*. cf. *schmidti*)))))). Although somewhat different from our phylogenetic proposal (Fig 1), due in part to a different set of species utilized, the findings of Aguilar-Puntriano et al. [66] validate the hypothesis that the lizards of the Tajzara Basin described in this present work represent a new species of the *L*. *montanus* group. The work of Jiménez-Robles and De la Riva [105] on an assemblage of four species of *Liolaemus* over a topographic and altitudinal gradients in the Tajzara Basin demonstrates that the lizards they identified as *Liolaemus* sp. which we here describe as *L*. *tajzara* sp. nov., is differentiated in its thermal ecology and use of microhabitats in respect to the three other sympatric species: *L*. *puna* (*alticolor-bibronii* group), *L*. *ornatus* (*boulengeri* group), and *L*. *orientalis* (*montanus* group).

This evidence from three independent lines of research–morphological, molecular, and ecological–firmly support the hypothesis that the lizards described as *L*. *tajzara* sp. nov. belong to a new species for Bolivia and for the *L*. *montanus* group and which in the past had been confused with *L*. *islugensis* and *L*. *signifer*.

## Appendix

*Liolaemus annectens* (n = 15): PERU: Arequipa: Sumbay, MUSA 4114, 4265–4266; Caylloma, 4344–4348; MUSA-CSA 1591–1597.

*Liolaemus chlorostictus* (n = 61): BOLIVIA: Potosí: Sur Lipez: Khastor, CBF 1062. ARGENTINA: Jujuy: Rinconada: way to Mina Pirquitas, FML 02284 (Holotype), 1510 1/17, 1522 1/21, 01515/1–14 (Paratypes); Mina Pirquitas FML 18225–232.

*Liolaemus erguetae* (n = 10): BOLIVIA: Potosí: Sur Lipez: Pena Barrosa, CBF 728–735, 1293 (holotype), 1295 (paratypes).

*Liolaemus etheridgei* (n = 17): PERU: Arequipa: Cabrerías: Cayma, MUSA 501; cerro Uyupampa, Sabandia, MUSA 549–554; Riparian mountain of Quebrada de Tilumpaya Chiguata, Pocsi 1113–1114, 1116, 1264–1268, 1353; Annex to Yura Viejo, Yura, MUSA 1229.

*Liolaemus famatinae* (n = 33): ARGENTINA: La Rioja: Famatina, FML 232; Cueva de Pérez, Nevados de Famatina, FML 1720/1–32.

*Liolaemus fittkaui* (n = 2): BOLIVIA: Cochabamba: Tiraque, FML 16121–16122.

*Liolaemus forsteri* (n = 16): BOLIVIA: La Paz: Murillo: Alto Achachicala, CBF 2485, 2705, 2707–2708, 3117–3118, 3123–3126; La Paz: Murillo: Milluni, CBF 2716–2717, 3053–3055, 3057.

*Liolaemus griseus* (n = 3): ARGENTINA: Tucumán: Tafí del Valle: Vega Mataderos, Vacahuasi, FML 1354; Cerro Lomo Ballena, FML 1582; Cerro Negrito, FML 1586.

*Liolaemus huacahuasicus* (n = 12): ARGENTINA: Catamarca: Andalgalá: Filo los Heladitos, FML 1224; Santa María: El Cerrillo, FML 486–487; Cerro El Overo, Nevados del Aconquija, FML 665; Filo Colorado, FML 674–675; Nevado del Candado, FML 677; Nevado de las Animas, FML 885; Tucumán: Tafí del Valle: Laguna El Negrito, FML 66 (Paratypes); Cerro El Negrito, FML 203; FML 469–470 (Paratypes).

*Liolaemus islugensis* (n = 18): BOLIVIA: Oruro: Atahuallpa: Chipaya, CBF 2687, 2690, 2851; Oruro: Atahuallpa: Huaylliri, CBF 2692–2693; Oruro: Atahuallpa: Lonchihuaylla, CBF 2559, 2622–2623; Oruro: Atahuallpa: Yunguyo, CBF 2581, 2625. CHILE: Region I: Isluga, FML 25958–25965.

*Liolaemus jamesi* (n = 10): BOLIVIA: Oruro: Sajama: Papel Pampa, CBF 1913. CHILE: Region I: Salar de Coposa, FML 28890, 28900–28901; Región XV: Parinacota, FML 1775, FML 28899; Chungará lake, FML 28855–28858.

*Liolaemus montanus* (n = 9): ARGENTINA: Catamarca: Ambato: El Rodeo, FML 279, FML 289; El Manchao, FML 214, FML 908–910, 981–982, FML 1723.

*Liolaemus orientalis* (n = 52): ARGENTINA: Jujuy Province: Humahuaca Department: Route to the Laguna Blanca, FML 928 (4), 949 (2); Chorcán, FML 930, 938–939, 944; Route to the Laguna Blanca, FML 949 (2); Laguna Leandro, West of Chorcán, FML 1456 (4); Mina Aguilar, FML 1537 (2); Quebrada Tonocote, FML 2035 (14); Tablayo, West to the Chaupi Rodeo, FML 2104 (6); Jujuy: Humahuaca: Laguna Leandro: FML 1877, 18192–18197; Camino a Mudana desde Uquía, FML 18211–18217.

*Liolaemus orko* (n = 9): ARGENTINA: Catamarca: Tinogasta: La Lagunita, Sierra de Fiambalá, FML 18416 (Holotype), FML 18417–18421 (Paratypes), MCN 2130–2131 (Paratypes), Las Pampas, Campo Potreritos, FML 1911.

*Liolaemus pachecoi* (n = 7): BOLIVIA: Potosí: Sur Lipez: Laguna Colorada, FML 2788 (Paratype). Pena Barrosa, CBF 743, 1057 (Holotype). CHILE: Region I: Quebrada del Inca, FML 28835–28836; Puquios, FML 28837, 28894.

*Liolaemus pleopholis* (n = 26): BOLIVIA: Oruro: Sajama, CBF 1866–1870; Junthuma, CBF 1887; 1895, 1910; Quilhuiri, CBF 1885–1886, 1909, 1911, 1914; Cosapa CBF 3714–3716, 3721–3722. CHILE Region XV: Pampa Chucullo, FML 26020–26027.

*Liolaemus pulcherrimus* (n = 17): ARGENTINA: Jujuy: Humahuaca: Mudana, FML 1961, 2184 (Paratypes); Ruta a Mudana, desde Uquía, FML 18221, 18238–18249; 24km east from Uquía, FML 18285, 18213.

*Liolaemus puritamensis* (n = 12): ARGENTINA: Jujuy: Rinconada, FML 18150. BOLIVIA: Potosí: Sur Lipez: Khastor, CBF 736–737, 1054, 1059, 1061; Río Blanco, CBF 744; Quetena Chico, CBF 1926. CHILE: Region II: Baños de Puritama, FML 28955–28958.

*Liolaemus schmidti* (n = 15): BOLIVIA: Potosí: Sur Lipez: Pena Barrosa, CBF 715–727. CHILE: Region I: Puquios, FML 25978, 25983.

*Liolaemus signifer* (n = 20): BOLIVIA: La Paz: Tiahuanaco, CBF 1985; Tacaca, CBF 468, 471–475; Queruni, CBF 1765; Calacoto, CBF 2252–2261. PERÚ: Puno: Titicaca lake, FML 1434, FML 1557.

*Liolaemus tajzara* sp. nov. (n = 32): BOLIVIA: Tarija: Avilez, CBF 1766–1768, 1770–1773, 1778–1783, 1788–1790, 4608–4617; FML 3581, 3584, 35871–35874.

## Supporting information

S1 Dataset Phylogenetic analysis. (TXT)

S1 Table Statistical analysis. (XLS)
